# Working Differently or Not at All: COVID-19’s Effects on Employment among People with Disabilities and Chronic Health Conditions

**DOI:** 10.1177/07311214211012018

**Published:** 2021-05-06

**Authors:** Michelle Lee Maroto, David Pettinicchio, Martin Lukk

**Affiliations:** 1 University of Alberta, Edmonton, AB, Canada; 2 University of Toronto, Toronto, ON, Canada

**Keywords:** disability and society, inequality, poverty and mobility, COVID-19, work

## Abstract

The COVID-19 pandemic has drastically changed employment situations for workers everywhere. This is especially true among people with disabilities and chronic health conditions who face greater risks in contracting COVID-19 and experience larger disadvantages within the labor market. Drawing from original data gathered through a national online survey (*N* = 1,027) and integrated set of virtual interviews (*N* = 50) with Canadians with disabilities and chronic health conditions, our findings show that although the pandemic has not directly led to job losses for most people with disabilities and chronic health conditions, respondents who have lost employment due to COVID-19 are struggling. Even though employed workers have been faring better, half were concerned about losing their jobs within the next year, and these concerns were more prevalent among part-time and non-union workers. Our findings emphasize the potential for growing economic insecurity as the pandemic continues to wreak havoc on employment situations among marginalized groups.

## Introduction

Boom. Because what happened is when we got back, they pretty well spun on us that we were getting paid minimum wage and very little commission. Because before then, we were getting paid commission. I lost pretty well 75% of my income. (Glenn)

Glenn,^1^ who is Deaf and has a heart condition, described his return to a sales job in a furniture store. He is facing a great deal of uncertainty as furniture manufacturing slows, and the retail brand he works for contemplates the possibility of shutting down branches due to lower sales. Although he remains employed, Glenn is earning a much lower income. As a result, he is relying on his savings and Canada’s taxable wage subsidy known as the Canada Emergency Response Benefit (CERB) to make ends meet. This has taken a toll on his financial situation. As Glenn explained, “when you see your bank account dwindling here and it’s not increasing . . . you just try to figure out and find, ‘Okay, how many months can I keep doing this?’”

Throughout the COVID-19 pandemic, this question has been on the minds of millions of Canadians. Growing financial insecurity, even among those who continued to work, remains a concern. It is especially salient among people with disabilities and chronic health conditions, who in addition to being at the greatest risk for developing complications if they contract COVID-19 (Centers for Disease Control and Prevention 2021), also confront large labor market barriers that contribute to un- or under-employment, low wages (Blanck et al. 2007; Maroto and Pettinicchio 2015, 2020; Morris et al. 2018; Wall 2017), and clustering in precarious work (Jones 2008; Maroto and Pettinicchio 2014a). In addition, people with cognitive disabilities and multiple disabilities are far more likely to experience negative labor market outcomes than individuals with respiratory and heart problems and physical disabilities (Jones 2011; Zwerling et al. 2003) in part because of their employment in precarious food preparation and service sector jobs (Maroto and Pettinicchio 2014a; Wall 2017).

These employment inequalities are exacerbated by crises like the COVID-19 pandemic. This is the case in countries like the United States, where pandemic countermeasures were often delayed or very limited, and in countries like Canada, where governments acted more swiftly (Béland et al. 2021). By March 2020, Canadians faced severe travel restrictions as well as lockdowns and business closures. Consequently, weekly work hours among the working-age population decreased by 32 percent in March and April 2020 in Canada (Lemieux et al. 2020). To help address employment losses and rising financial insecurity, the federal government enacted CERB, a taxable Can$2,000 monthly subsidy for those losing employment during the pandemic.

Although layoffs increased across Canada, women, racial minorities, and lower income workers have faced higher rates of unemployment and greater losses of income and work hours (Lemieux et al. 2020; Qian and Fuller 2020). Even workers who have remained employed have seen their work drastically change. In both Canada and the United States, an estimated 40 percent of the labor force was working from home full-time by May 2020 (Bloom 2020; Statistics Canada 2020b). Although many working from home must now contend with a new work–life balance (Schieman and Badawy 2020) while learning new ways to approach their jobs, people with disabilities and chronic health conditions face additional challenges when it comes to transferring their workplace accommodations to their home. Many, for example, require specialized equipment and software that might not be available for a home office.

Those who continue to work outside their home—“essential workers” in the service sector like grocery store and pharmacy workers—are facing additional work hours, longer shifts, and less flexibility, changes that vary across occupations. Many people with disabilities are clustered in food preparation, service/retail, and manufacturing sectors among the most affected by COVID-19 (Maroto and Pettinicchio 2014a; Mather and Jarosz 2020). Many others have been furloughed or laid off. Others, like Glenn, were asked to come back to work but under strict social distancing measures and sanitization protocols. Unlike those in essential service sector jobs, workers like Glenn are also facing reduced hours and in turn, reduced income.

Employment is one of many areas in which the global health pandemic highlights the devastating effects of accelerating structural disadvantage on the lives of marginalized peoples. Using original data gathered through a national online survey and integrated set of virtual qualitative interviews with Canadians with functional limitations, chronic illnesses, and other underlying health conditions, this paper examines the employment impacts of COVID-19 on people with disabilities and chronic health conditions with an emphasis on the potential for growing economic insecurity. We address the following questions:

**Research Question 1:** How has the pandemic affected employment among people with disabilities and chronic health conditions?
**Research Question 2:** What has employment (or lack thereof) specifically meant for their economic situations during the pandemic?
**Research Question 3:** Among employed workers, how has the nature of their work changed and what is their future outlook on their employment situation?

In answering these questions, we speak directly to individual responses to COVID-19 and address how related measures have further compounded extant economic inequalities among already disadvantaged groups. Our findings show that although the pandemic has not directly led to job losses for most people with disabilities and chronic health conditions, a group with already low employment levels, those who have lost their jobs due to COVID-19 are struggling financially. Sixty percent of these respondents reported worsening financial situations and over 70 percent reported that COVID-19 affected their ability to pay down debt, make mortgage or rent payments, pay utility bills, purchase groceries, or contribute to savings to a moderate or great extent.

Among those who kept working, two-thirds of respondents saw their jobs fundamentally change either by shifting to remote work or experiencing increased amount of work. Even though employed workers have been faring better than those without work in the pandemic, employment is not always a path to security, particularly among people with disabilities and chronic health conditions. Half of employed respondents were concerned about losing their jobs within the next year, and these concerns were more prevalent among part-time and non-union workers. As many places muddle through second and third waves of the pandemic, these findings highlight potentially dire economic consequences given that people with disabilities already experience some of the highest rates of economic insecurity and poverty.

## Disability and Health as Axes of Inequality Limiting Employment

Like gender, sexual identity, race, ethnicity, and class, disability and health status influence experiences, social interaction, and well-being (Markus 2008). Disability, chronic illness, and perceived negative health often receive a lower status value through ascriptive processes (Link and Phelan 2012; Phelan et al. 2014), affect interpersonal interactions, and influence access to resources (Kemper 1974; Ridgeway 1991), making them stigmatizing master statuses that continue to disadvantage and oppress historically marginalized communities. Employment exclusion is by far one of the most acute barriers (Maroto and Pettinicchio 2015, 2020; Shier, Graham, and Jones 2009); half of all complaints to the Canadian Human Rights Commission are disability-based and many are labor market–related (Government of Canada 2017).

People with disabilities and chronic health conditions have exceptionally low employment rates in all industrialized countries (World Health Organization [WHO] and World Bank 2011). Chronic health conditions including diabetes and cardiovascular diseases lead to lower employment rates because these individuals exit the labor market earlier at significantly higher rates as a result of their health status (Organisation for Economic Co-operation and Development/European Union 2016). In the United States, the employment rate among working-age (16–64 years) adults with disabilities hovers around 30 percent (U.S. Bureau of Labor Statistics 2020). In the United Kingdom, it is about 54 percent (Powell 2020), and in Canada, the disability employment rate is just under 50 percent (Morris et al. 2018). Rates are similar across other industrialized countries (WHO and World Bank 2011). However, in the Global South, disability employment rates are closer to 10 to 20 percent (Hanass-Hancock and Mitra 2016; Mizunoya and Mitra 2013).

Work is not always a path out of poverty, either. Wages among people with disabilities remain low (Maroto and Pettinicchio 2014b). When they do work, people with disabilities are often segregated into low-paying, precarious non-union jobs in the food and service sectors (Jones 2008; Kaye 2010; Maroto and Pettinicchio 2014a), where workers are more likely to experience unmet needs due to a lack of necessary accommodations in turn leading to work interruptions and low earnings (Pettinicchio and Maroto 2020; Shuey and Jovic 2013). Outcomes may be further compounded by unequal and discriminatory wage policies and structures negatively affecting workers with disabilities and chronic health conditions (Kaye, Jans, and Jones 2011; Pettinicchio and Maroto 2020; Schwochau and Blanck 2000; Stein 2003).

A prevalent concern among workers with disabilities and chronic health conditions is the lack of workplace accommodations that facilitate maintaining employment and continuous earnings (Burkhauser, Butler, and Kim 1995; Chow, Cichocki, and Croft 2014; Schur, Kruse, and Blanck 2005; Solovieva, Dowler, and Walls 2011). Both Canadian and American legislation mandate that employers and businesses have some duty to accommodate as a matter of rights. However, accommodations vary by the nature of disability, not all health conditions are legally considered to be disabilities, and the likelihood they are implemented varies by occupational sector, affecting employment interruptions and earnings (Lurie 2017).

To make matters worse, people with disabilities and chronic health conditions face a complex and often perverse system of benefits based on employment and earnings thresholds that contribute to economic disadvantage and inequality. For example, many Canadians with disabilities, already struggling to make ends meet with little-to-no savings (Maroto 2016), are discouraged in finding slightly higher earning employment, fearing they will lose benefits supplementing their meager earnings (Maroto and Pettinicchio 2020). In effect, they would be worse off with a better job and no government assistance, than in a bad job with assistance.

To summarize, due to various labor market barriers, employment rates remain low among people with disabilities and chronic health conditions. When available, work tends to be precarious and low-paying. This was the case even prior to the pandemic and recent evidence indicates that the pandemic is exacerbating these inequalities.

## COVID-19, Health, Disability, and Employment

With already limited resources, members of marginalized groups are more susceptible to illness and disease, face larger barriers in accessing appropriate health care, and are less able to weather the hardships created by disasters and economic downturns (Link and Phelan 2004). Evidence from previous crises bears this out. During the Great Recession, U.S. workers with disabilities were among the first to be laid off and saw much greater increases in unemployment than workers without disabilities (Fogg, Harrington, and McMahon 2010; Kaye 2010; Livermore and Honeycutt 2015). Between 2007 and 2013, U.S. workers with disabilities were 75 to 89 percent more likely to experience involuntary job loss than workers without disabilities (Mitra and Kruse 2016). Workers with chronic health conditions and health limitations likewise faced a much greater risk of job loss during the Great Recession than those without them (Reeves et al. 2014).

For people with disabilities and health conditions, the COVID-19 pandemic has compounded barriers in accessing health and social services, resulting in even greater susceptibility to the virus. Between March and July 2020, disabled people accounted for 60 percent of all COVID-19 deaths in England and Wales (UK Office for National Statistics 2020). Death rates also varied by gender and disability type, with men and women with more severe disabilities having age-standardized COVID-19 mortality rates of 240.84 and 169.89 per 100,000 persons in the population, respectively (UK Office for National Statistics 2020). In the United States, people with intellectual and developmental disabilities were at a higher risk of getting and dying from COVID-19 and developing other complications from COVID-19 (Turk et al. 2020).

In addition to the different illness risks, the recovery will be felt unequally across groups, highlighting the link between health and economic inequalities (Anderson, Bjorklund, and Rambotti 2019; Hill and Jorgenson 2018; Sharma 2020). Canadians who happened to be unemployed and looking for work as the pandemic hit faced a dramatic decrease in job vacancies, as high as a 50 percent decline in the number of jobs available (Bell and Blanchflower 2020; Jones et al. 2020). These individuals will therefore experience even longer periods outside the labor market.

For those who were working, recent crowdsourced data from Statistics Canada (2020a) show that 36 percent of respondents with disabilities experienced a temporary or permanent job loss as a result of the pandemic; more than half of participants reported difficulties meeting financial obligations. Job losses have been clustered in the low-paying food, service, and sales sectors, where disabled Canadians tend to work (Goddard 2020; Lemieux et al. 2020; Mather and Jarosz 2020), with perhaps only those considered “essential workers” in these sectors feeling relatively greater security (although not necessarily better working conditions).

Essential workers, although benefiting from employment, face additional risks when it comes to exposure to the virus. The risk of infection based on physical proximity to other workers and overall exposure to infections or diseases tends to be higher in occupations dominated by women and higher in low-income and service occupations (St-Denis 2020). Not only does this jeopardize workers’ health but getting sick can also lead to unemployment. Essential workers may also be working longer hours with less flexibility in scheduling, a change that can disproportionately affect people with disabilities and chronic health conditions. The jobs in question are often non-union jobs with fewer benefits that do not necessarily guarantee long-term financial security. Moreover, the sense of insecurity experienced in these jobs itself constitutes a source of stress that can have negative independent health and mental health effects (Grace 2020; Kahn and Pearlin 2006; Thoits 2010). Thus, the precarious jobs that people with disabilities and chronic health conditions disproportionately find themselves in further exacerbate health inequalities. The pandemic has made the precarity of work and health all the more salient to an already vulnerable segment of the population.

The nature of work is also changing during the pandemic and these changes have introduced additional inequalities. Some workers, primarily those in higher earning office-based occupations (e.g., professionals in the finance sector or in government and public administration) have shifted from office buildings to home offices (Gallacher and Hossain 2020). For some, this transition may only be temporary, but for others, employers may consider remote work as a more permanent arrangement (Deng, Morissette, and Messacar 2020). Working from home is also not without its conflicts, especially when childcare is limited (Qian and Fuller 2020), but having this option provides at least some protection to the spreading virus. Other workers, primarily those workers dubbed essential, are still required to leave their homes for work on a regular basis or face losing their incomes.

Outcomes throughout the pandemic have also been tied to broader government responses, primarily at the federal level. CERB is at the center of the bundle of policies intended to respond to the economic consequences of the pandemic in Canada. These kinds of benefits tied to broader social welfare policy arrangements can help buffer the negative health and economic impacts brought on by crises and shocks (Beckfield and Bambra 2016; Beckfield and Krieger 2009). Although welfare states play an important part in reducing health and socioeconomic inequalities among more vulnerable groups (Cambois et al. 2016; van der Wel, Dahl, and Thielen 2011; van der Wel et al. 2018), historically, crisis policy responses have benefited social groups unequally, increasing disadvantage for the most marginalized (Bitler, Hoynes, and Kuka 2017; Moffitt 2013). For example, Canadians with disabilities and chronic health conditions without employment prior to the current pandemic have been excluded from benefits like CERB. Even as CERB was rolled into Employment Insurance in fall of 2020, it continues to be of little help to people with disabilities and chronic health conditions.

In this vein, we address the employment effects of COVID-19 on people with disabilities (i.e., people with functional and activity limitations), chronic illnesses, and other underlying health conditions (e.g., heart disease, hypertension, diabetes, chronic respiratory diseases, and cancer)—a significant segment of the population that already experiences higher unemployment and poverty rates and often relies on income outside of the labor market. We speak directly to individual responses to COVID-19 and how related measures have further compounded extant economic inequalities among already disadvantaged groups.

Based on existing work on occupational segmentation into so-called “good” and “bad” jobs (Kalleberg, Reskin, and Hudson 2000) and more recent work on the impacts of COVID-19 on the labor market, we expect that individuals in “good jobs” will experience less insecurity during the pandemic. Workers in public sector, unionized jobs with security and benefits, who would have more likely transitioned to remote work, are also more likely to see less income disruption and experience less financial insecurity. Those working in “bad jobs,” such as those in the food and service sector that are non-unionized with few benefits and low earnings, prior to the pandemic will experience greater interruptions and greater risk of economic insecurity. At the same time, unemployed individuals and those seeking work prior to the pandemic will continue to remain unemployed with no income, likely relying on minimal provincial disability benefits but not eligible for federal COVID-19-related income supports like CERB and Employment Insurance programs.

## Data and Method

This paper uses data from a mixed-methods project that includes a national survey of Canadian adults with disabilities and/or chronic health conditions conducted in June 2020 and a follow-up set of interviews with survey participants conducted in August through November 2020, to examine how members of these groups have managed rapidly changing work conditions.

### Survey Data Analysis

Survey data come from a quota-based online survey administered by Qualtrics, an Internet-based survey company that uses paid research panels of respondents, from June 11–22, 2020. Data were collected via quota-based sampling to ensure that we obtained a sample representing all 10 Canadian provinces.^2^ Because it was unclear on which population characteristics we should base any additional quotas or weights (as there have been no other random surveys of Canadians with disabilities and chronic health conditions), we do not employ poststratification weights based on group characteristics (Bethlehem 2010; Schonlau et al. 2009). However, many of the characteristics of this group (e.g., age, gender, and education) mirror those for individuals sampled in the Canadian Survey on Disability and Canadian Community Health Survey. We note such comparisons in the discussion of descriptive statistics in Supplemental Appendix A.

The full survey includes 1,027 respondents aged 18 and older who reported having one or more disabilities or health conditions, as outlined in Supplemental Appendix A.^3^ We use these data when examining the relationship between employment situations and insecurity. For other analyses, we rely on survey results from 490 respondents with employment. Given that this is a unique dataset assessing relatively recent developments among vulnerable groups on which little data exist, much of the analysis is descriptive, exploring underlying trends and patterns. We focus on studying these patterns through sets of bivariate logistic regression models predicting experiences of insecurity, concerns regarding job loss, and changing work situations in relation to employment status and working conditions.

### Outcome Variables

We examine five variables as measures of COVID-19-related financial insecurity, employment precarity, and employment changes. We include a measure of *worsening financial insecurity* based on responses to the question, “Compared to 1 year ago, would you say your household’s financial situation is worse than it was, the same, or better than it was?” This variable indicates whether the respondent answered “worse.” We look at *COVID-19-specific effects on financial outcomes* with a variable that indicates whether COVID-19 affected a respondent’s ability to pay down debt, make mortgage or rent payments, pay utility bills, purchase groceries, or contribute to savings to a moderate or great extent. This variable combines the results of five separate variables regarding COVID-19’s effects for each of these areas.

Among employed respondents, *job loss concerns* indicates whether the respondent voiced concerns about job loss due to COVID-19 within the next year. It is coded as a binary variable indicating whether the respondent reported that they were somewhat or very concerned about job loss. We measure employment changes with two binary variables—whether the respondent now *works from home* for part or all of their work hours and whether the respondent has *taken on more work* due to COVID-19 distancing measures. Please see Supplemental Appendix A for descriptive statistics for all key variables.

### Predictor Variables

We study how work outcomes vary across respondents’ employment statuses and working conditions. *Employment status* includes eight categories: employed full-time; employed part-time; not working, looking for work (unemployed); not working, homemaker; not working, in school; not working, retired; not working, unable to work due to COVID-19; and not working, unable to work for other reasons.^4^ We also look at whether respondents *had applied for CERB*, the federal wage subsidy for people whose employment had been affected by COVID-19.

Working conditions refer to occupation, union membership, and part-time work. We coded *occupation* based on open-ended questions where respondents provided information about their current occupation. The occupational variable includes the following broad categories: management occupations; business, finance, and administration occupations; natural and applied sciences and related occupations; health occupations; occupations in education, law and social, community and government, art, recreation, culture, and sports; sales and service occupations; trades, transport and equipment operators, natural resources, manufacturing, and utilities; and military and other occupations.^5^ *Union membership* and *part-time work* are dichotomous variables based on respondent self-reports.

### Interview Data Analysis

To supplement our survey data with more nuanced accounts of people’s economic situations throughout the pandemic, we draw from 50 in-depth phone interviews with respondents surveyed. In all, 506 survey respondents requested to be contacted for follow-up interviews. We sampled 100 of these respondents; narrowed this list down to ensure that people with different disabilities, health conditions, and other characteristics (i.e., age, gender, race) were represented in our interview sample; and then contacted respondents on this list. We offered additional Can$30 Amazon gift cards to those wishing to participate.

Echoing findings from the survey data, half (25) of the interview respondents were employed either before or during the pandemic. Although we also discuss the situations of respondents who struggled to find work before the pandemic, we primarily rely on interviews with the 25 employed respondents for our analyses. Please see Supplemental Appendix B for more details about interview respondents.

The interview data cover an ever-shifting pandemic context beginning with more optimistic outlooks in August 2020 as COVID-19 cases were declining steadily in Canada, to more recent fears as Canada entered its second wave in mid-September. Interviews ranged from 12 to 60 minutes in length with a mean length of 33.9 minutes and a median length of 33.4 minutes. Audio files were transcribed and coded in batches by a team of research assistants. An initial subset of transcripts was coded by each team member to generate a preliminary coding scheme, derived deductively based on the study’s research questions and inductively based on additional emergent themes in the data (Deterding and Waters 2021). Through multiple consultations, codes developed by team members were compared and reconciled to achieve a final coding scheme, which, after pre-testing on an additional subset of interviews to ensure consistent application, was used to code all 50 interviews. These codes focused on the areas of employment, work situations, and financial security. To supplement our quantitative analysis, our findings discuss responses from interview subjects whose narratives exemplify the major themes that emerged in this analysis.

## Findings

Although the pandemic has not directly led to job losses for most people with disabilities and chronic health conditions, a group with already low employment levels, our findings show that those who have lost employment due to COVID-19 are struggling more than others. Survey respondents without employment were more likely to report worsening financial situations, which many further described throughout the interviews. Although employment provided a buffer to financial insecurity, employed respondents still reported concerns about job loss and described worsening employment situations, characterized by increased precarity combined with lower wages.

### Employment Situation

Many respondents with disabilities entered into the pandemic without employment and without federal COVID-19-related income supports, like CERB. As shown in Table 1, at the time of the survey, 35.2 percent of respondents were employed full-time and another 12.6 percent were employed part-time, 4.4 percent were unemployed, and 47.9 percent were not in the labor force, which included homemakers, students, retired persons, and people unable to work due to COVID-19. In this sample, 8.8 percent of respondents reported that they were unable to work due to COVID-19.^6^ June 2020 Labor Force Survey data show higher employment rates among the larger population (Supplemental Appendix D). In addition, 21.5 percent of respondents applied for CERB (the number is closer to 30 percent among all Canadian adults) with another 5.2 percent indicating plans to do so in the future. Notably, however, respondents who were not employed before the pandemic are unlikely to qualify for CERB, often relying almost exclusively on a small one-off payment and provincial disability benefits which vary considerably.

**Table 1.** Employment Situation and CERB Receipt, All Respondents.

Employment Situation	Frequency	Percentage
Detailed employment status		
Employed full-time	361	35.15
Employed part-time	129	12.56
Not working, looking for work	45	4.38
Not working, homemaker	28	2.73
Not working, in school	33	3.21
Not working, retired	256	24.93
Not working, unable to work due to COVID-19	90	8.76
Not working, unable to work for other reasons	85	8.28
Applied for CERB		
Have not applied for CERB and have no plans to	753	73.32
Have not applied for CERB but plan to	53	5.16
Have already applied for CERB	221	21.52

*Source.* The 2020 COVID-19 Response Survey of People with Disabilities and Health Conditions, *N* = 1,027 adults.

*Note.* CERB = Canada Emergency Response Benefit.

Employment struggles were also common among interview respondents with half reporting being employed at the time of their interviews. For example, Esther, who has stage 4 osteoarthritis and depression, tried looking for work as a personal support worker for which she received training. But as she explained,

I am unable to do the lifting, etcetera. I have been looking for jobs with low degree work, but it’s not available for me. Everybody wants full-time work, and I don’t think I can do that right at this point.

Maryam, an academic coach who reported emotional disabilities, was applying for jobs just as COVID-19 hit. The places she applied to were no longer looking for employees as a direct result of the pandemic leaving her unemployed and without steady income.

Others, like Glenn, were involuntarily laid off on a temporary basis and made use of CERB during their unemployment. Glenn then returned to work at significantly reduced earnings. Antoine, a school bus driver with diabetes, and high blood pressure and cholesterol, was furloughed early in the pandemic and also made use of CERB. He recently returned to work driving a bus once a week and, at the time of the interview, was expecting to return to his regular schedule when schools reopened.

Although some laid off respondents like Glenn returned to work, others who were laid off, especially in sectors hard hit by the pandemic, were not working at all and indicated that they may not be able to return to their previous job. For example, Daniel worked in sales at a hotel chain. Daniel has HIV and was making use of extended government health benefits to cover the costs of his medication. Not knowing whether he will ever be called back to work, he is now living entirely on CERB and provincial disability benefits.

Some saw the dire labor market situation as an opportunity to stop looking for work and enroll in higher education. For example, both Cheyenne and Ramona believed pursuing higher education would help them in the labor market once it recovers. Cheyenne, an Indigenous woman with osteoporosis, obessive-compulsive disorder (OCD), and an eating disorder, worked in a café that closed because of COVID-19. She is receiving CERB as well as disability benefits and will be enrolling in a human resource management certificate program, hoping to get out of the food/retail sector.

For Ramona, who has endometriosis, having an in-person job during the pandemic is not an option. Interviewed just when Canada entered the second wave of COVID-19, she explicitly cited renewed concerns about contracting the virus, stating, “there’s this conflict between wanting to keep myself safe, but also wanting to work.” Before the pandemic, Ramona worked as a barista but would rather improve her credentials so she can get a better job post-pandemic. As Ramona noted,

I’m trying to get out of the service industry and into something administrative, so hopefully, there would be an increase in income then, and then that trickles down into more savings and being able to pay off more debt. So, I’m optimistic about that.

It is clear from respondents’ employment situations and the percentage of respondents applying for CERB that COVID-19 has directly affected employment for people with disabilities and chronic health conditions, as it has the larger population. Many of their struggles are also exacerbated by their specific disabilities and health conditions, which kept many out of work and limited the work they could do during a pandemic. But, COVID-19 is not only directly affecting employment security—we also find that even respondents who have remained in their jobs experienced increasing financial insecurity.

### Employment and Financial Insecurity

**Figure 1.**
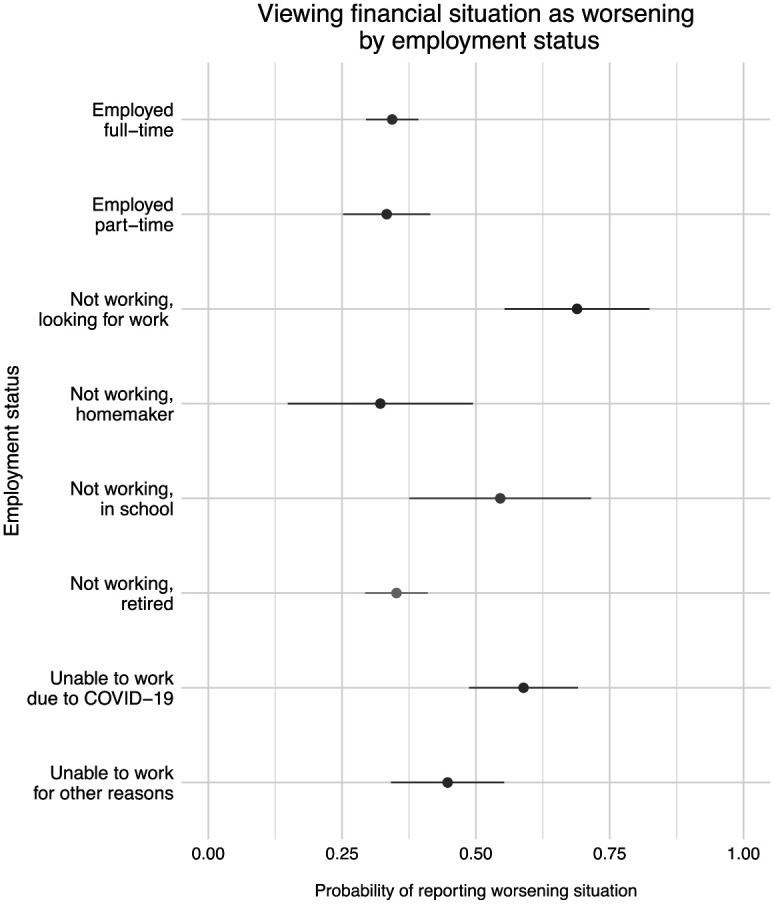
Respondent views on current financial situation compared with previous year by employment status. *Source.* The 2020 COVID-19 Response Survey of People with Disabilities and Health Conditions, *N* = 1,027 adults. *Note.* Point estimates and 95 percent confidence intervals.

Financial insecurity tended to be lower among respondents with full- or part-time employment, those who were retired, and those who described themselves as homemakers. Figure 1 presents proportions of respondents, by employment status, who viewed their financial situations as worsening compared with the previous year.^7^ As shown in this figure, 34.4 percent of full-time workers, 33.3 percent of part-time workers, 32.1 percent of homemakers, and 35.1 percent of retired persons reported worsening financial situations. This is compared with 68.9 percent of unemployed respondents, 54.5 percent of respondents in school, 58.9 percent of respondents unable to work due to COVID-19, and 44.7 percent of respondents out of work for other reasons.

**Figure 2.**
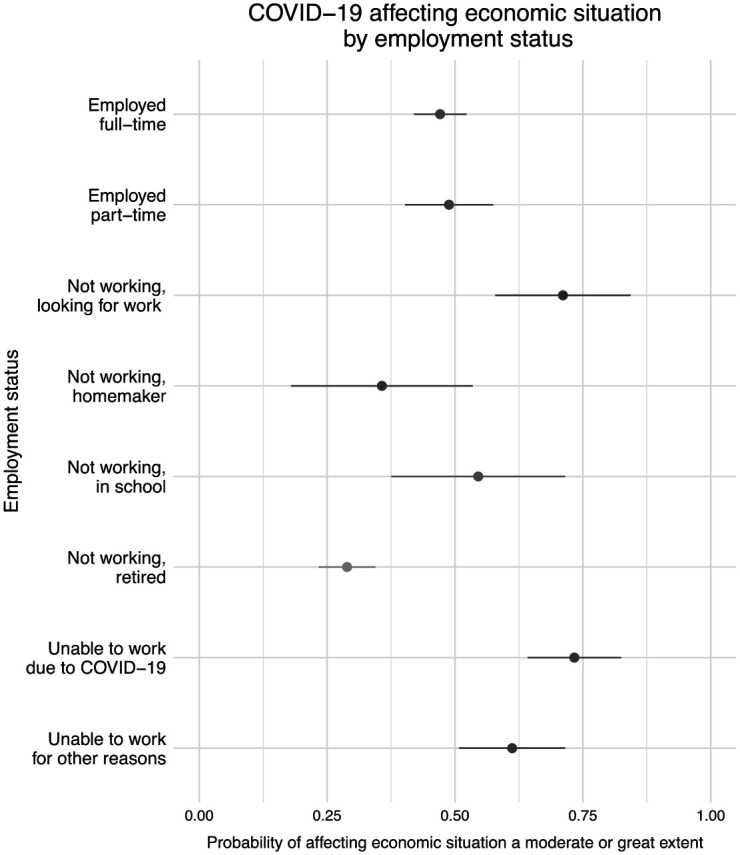
Respondent reports of COVID-19 leading to negative economic and financial effects by employment status. *Source.* The 2020 COVID-19 Response Survey of People with Disabilities and Health Conditions, *N* = 1,027 adults. *Note.* Point estimates and 95 percent confidence intervals.

In addition to worsening financial situations, 47.2 percent of respondents reported that COVID-19 affected their ability to pay down debt, make mortgage or rent payments, pay utility bills, purchase groceries, or contribute to savings to a moderate or great extent, but this varied by employment status, as shown in Figure 2. Retired respondents and homemakers were the least likely to report negative economic effects due to COVID-19, followed by employed respondents. Respondents who were not working and looking for work and those who were unable to work due to COVID-19 were far more likely to indicate that the pandemic led to negative economic consequences. Among those looking for work, 71.1 percent reported that COVID-19 affected one or more economic outcomes to a moderate or great extent, and 73.3 percent of respondents unable to work due to COVID-19 also reported negative economic effects.

CERB has become an important option for respondents in these categories, but only when respondents were eligible for this income support, which was generally not the case for people with disabilities and chronic health conditions. Several respondents pointed to CERB in helping them get by as they remain out of work directly because of the pandemic. Glenn, for example, used CERB to supplement his reduced income. For others, it was their only source of income. While Daniel, a Toronto resident in an expensive housing market, found CERB critical, he also noted its limitations, saying,

It’s really not a lot to survive on. For myself and for many people. I mean, my rent is $2,100 a month and the CERB is $2,000. So, once you factor that in plus all your other bills, it’s pretty scary. It’s a pretty precarious place to be.

Others described how CERB helped supplement other income. Antoine noted that while he worries about what the future holds, he has not encountered significant financial problems and has kept up with all payments. What helped Antoine weather the economic storm was a combination of savings, continued but reduced pay from his employer, and CERB. As Antoine explained,

I expected some money in the bank. I was getting paid partially by the school bus company during the layoff and CERB gave me the balance . . . mind you, I was squirreling some money away because I know it has to be taxable.

Cheyenne directly attributed her current ability to stay afloat to CERB. She noted,

with CERB I am earning about as much as I would have if I was working full time, if the pandemic hadn’t occurred. . . . I was already in support of universal basic income, but I feel like it’s even more important now.

Those without CERB and without employment income tended to be in much worse shape. Esther, who only receives meager provincial disability supports, said, “I live each day on the edge, wondering if I can make ends meet tomorrow and see if I can feed myself type of thing.” She also relied on various community supports and services, which became inaccessible during the pandemic, jeopardizing her housing. She explained,

I’m scared about that because I do need the help, very much so. I need the community services. Even housing that I used to have been able to get help through they’re no longer there. It’s sad what COVID’s done to everybody.

Interestingly, like Cheyenne, Esther remained hopeful that “the CERB thing actually gets put forth as a mandatory income because I can’t even make ends meet.” This may reflect talks over the last few years by the federal government to experiment with a basic minimum income on which CERB is in many ways modeled (Prince 2020; Snell 2020; Willms and Montgomery 2020).

Employment and government supports clearly offer some protective benefits against insecurity. However, despite continuing income from employment, more than a third of employed respondents indicated that their financial situations had worsened compared with the previous year. Almost half of employed respondents still found that COVID-19 limited their ability to make payments or put aside money in savings, indicating that, for many, employment simply was not enough to stave off financial insecurity.

### Financial Insecurity among Workers

Among employed respondents, their levels of job security varied across work characteristics and occupations. In total, 50.8 percent of employed respondents reported that they were concerned that they might lose their jobs within the next year and 40.6 percent were concerned about losing their job in the next month. These rates were much higher than those in the larger population where only 16 percent feared job loss within the next month in May 2020 (Supplemental Appendix D).

**Figure 3.**
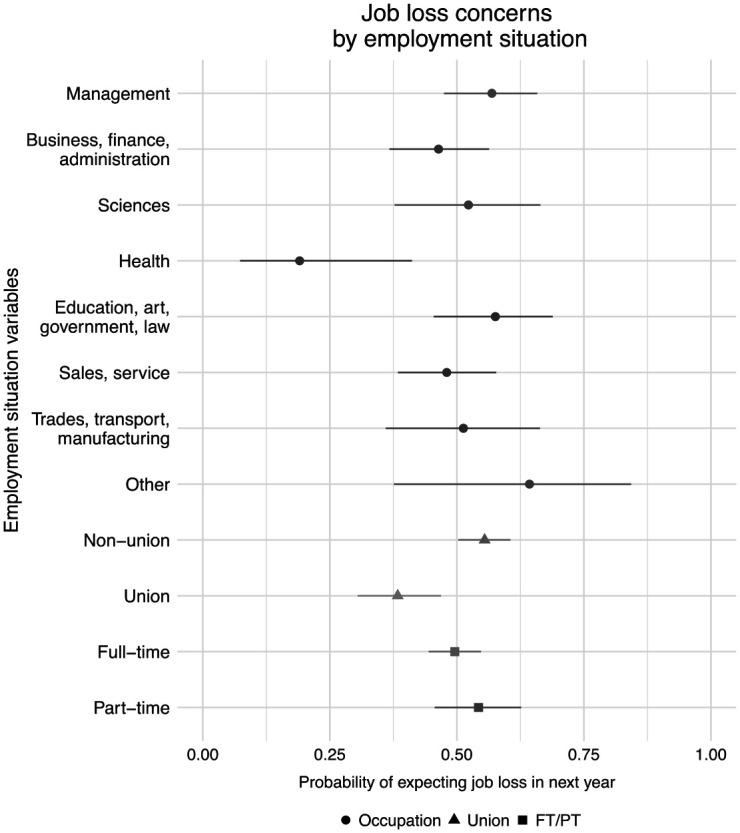
Respondent concerns about job loss in the next year by employment characteristics. *Source.* The 2020 COVID-19 Response Survey of People with Disabilities and Health Conditions, *N* = 490 adults with employment. *Note.* Point estimates and 95 percent confidence intervals.

As shown in Figure 3, these concerns were higher in certain occupations and among non-union workers. Compared with other occupations, health care workers were the least likely to be concerned about job losses with only 19.0 percent of respondents indicating some concern. Although few differences were present between respondents in full-time and part-time work, union membership tended to be a strong predictor of job security. Among workers in union jobs, 38.3 percent were concerned about job losses compared with 55.5 percent of non-union workers.

The pandemic directly changed the nature of many respondents’ jobs. For some, this also meant a loss of income. For example, Timothy, a sports instructor with severe asthma, continued to work from home during the pandemic but with far fewer hours. Although Timothy did not carry a lot of debt, he did not feel financially secure with reduced income. He says,

It’s been a big hit. I’m worried about that, how long it’s going to last for. I’ve had to use some of my savings, and like I said, I’ve had to cut back in as many areas as possible, and so yeah, until we get back, I’m not going to feel comfortable.

Similarly, Derek, an educational consultant for international students who has psoriatic arthritis and takes medication that suppresses his immune system, continued to work during the pandemic. But, with fewer international students coming into Canada, “a lot of business went away once the lockdown happened in March and it hasn’t come back . . . my revenue has virtually gone to nothing. So yes, impacted, I would say, on a really high scale.” Although a dual-income household, he noted that, their income is still “down to less than half . . . I still have a big mortgage to pay off, so that’s my biggest concern.”

For the most part, respondents with “good jobs,” particularly those that provided enough income to also allow them to save money, were far less likely to raise concerns about the negative impacts of COVID-19 on their future financial situation. Donald, for example, seemed very confident that the markets will return to normal. Similarly, Evan reported that he will come out ahead by investing savings now in the stock market which will eventually rebound. Interestingly, both noted that they are financially better off than most during the pandemic. Jasmine, a bank branch manager with severe depression, indicated that her job and income were not at all affected by the pandemic. While she is concerned about the future as the pandemic continues to unfold, she and her husband (also employed) are able to manage during the pandemic.

Government and unionized respondents also appeared to have greater protections, mirroring survey findings. Reagan, a government employee who has asthma and diabetes and is in remission from ovarian/uterine cancer, also holds a part-time job in a grocery store. When asked about her financial situation, her response was simply “I’m a single mother with two kids, right?” She attributed her second job as helping her get by when she lost her government job in 2008 (which she subsequently returned to in 2012). Even with employment insurance and government employee benefits, she had little income, reduced savings, and eventually foreclosed on her home. Nonetheless, because she had been in her government job for years prior to COVID-19, she believed she would be little affected by the pandemic, despite her prior financial devastation. Even individuals who were struggling to pay down debt, like Jean-Marc, a government employee, noted how a good job allowed him to continue to work, contributing to his sense of security, saying, “we’re unionized, so in our convention [collective bargaining agreement] . . . we’ll probably also have a small raise this fall. So, yeah. I think it’s going to be better.”

Other respondents were in occupations that allowed for paid leave, making it easier for them to avoid exposure to COVID-19. Donald, for example, is a postal worker with high blood pressure who voluntarily took time away from work. He noted the increased workload faced by postal workers and does not plan to return until things calm down.

Thus, we found that economic experiences varied among employed individuals based on the nature of their jobs and their pre-pandemic financial situations. Importantly, many employed individuals, even those in well-paying occupations with job security, still expressed concerns about their financial outlook. Several respondents reported that the pandemic served as a reminder that they were only a few paychecks away from not meeting their financial obligations while struggling to save for a rainy day, perhaps reflecting a more widespread reality among Canadian workers.

### Changing Work Conditions

**Figure 4.**
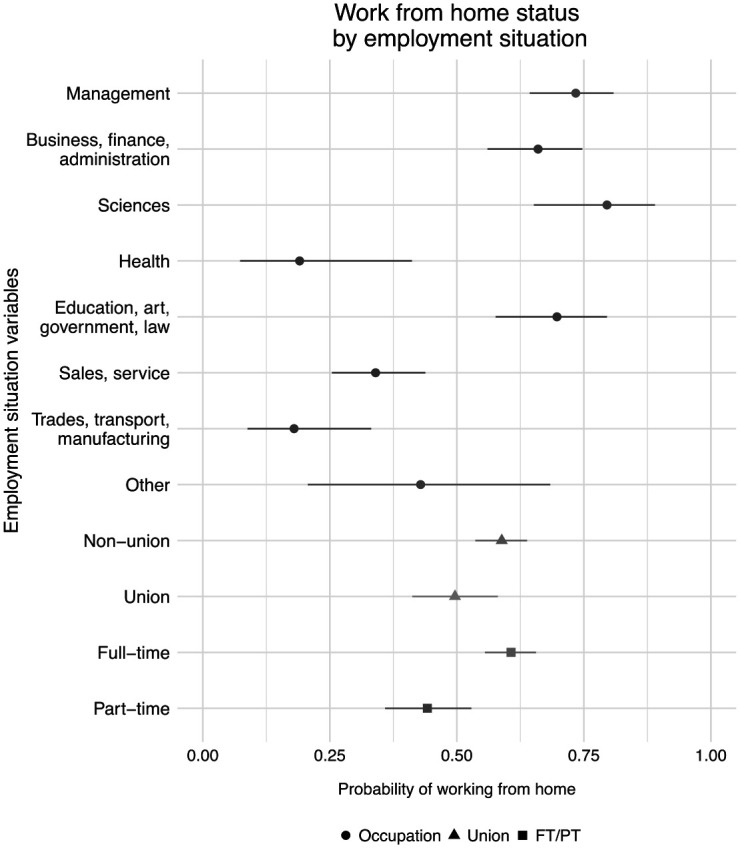
Respondents working from home by employment characteristics. *Source.* The 2020 COVID-19 Response Survey of People with Disabilities and Health Conditions, *N* = 490 adults with employment. *Note.* Point estimates and 95 percent confidence intervals.

In addition to concerns about job losses, workers also experienced changes in how and when they worked. Most employed respondents were able to transition to working from home, with 56.3 percent of respondents working completely or partly from home, which reduces exposure to the virus. Figure 4 shows how the probability of working from home varied across occupations, union job status, and work hours.

Respondents working in management jobs; jobs in business, finance, and administration; science-related jobs; and jobs in education, art, government, and law—primarily white-collar occupations—were the most likely to report working from home. Seventy to eighty percent of respondents in these jobs were able to work from home during the pandemic. Although they experienced higher levels of job security, health care workers were among the least likely to work from home with 19.0 percent working partly or fully from home. Workers in sales and service occupations and those in trades, transport, and manufacturing jobs were also less likely to work from home, which is generally expected due to the requirements of their jobs.

Few differences in work from home status were present across union and non-union members. However, full-time work was associated with a greater probability of working from home. Approximately 60.7 percent of full-time employees were able to work from home compared with 44.2 percent of part-time workers.

These comparisons show that the ability to work from home is not available to all workers. Those who could not work from home, especially those in retail and food jobs, like Cheyenne and Ramona, and those in sectors heavily affected by the pandemic, like Daniel, have come to face a great deal of uncertainty in the labor market and have to rely on other sources of income to get by.

Those who now worked from home did not see disruptions in their employment income but several respondents did report challenges in adapting to these new arrangements. For example, Evan has asthma and works in the financial sector. Although he noted the psychological toll working from home has taken, namely leading to a sense of isolation, he has been “glued” to his phone and computer which he feels makes the days go by faster. Others were already working from home as part of their disability-based accommodation. Reagan had returned to a modified work arrangement in her government job in 2012 after receiving cancer treatment and continued to work from home prior and during the pandemic. She found working from home a bit of a struggle at first but, as she explained, “when COVID hit, I was the least impacted in my department, because I was already set up for working from home. Everybody else was having problems, but I’m okay with it.” However, because of her experience working from home, she took on more work from her colleagues who struggled.

**Figure 5.**
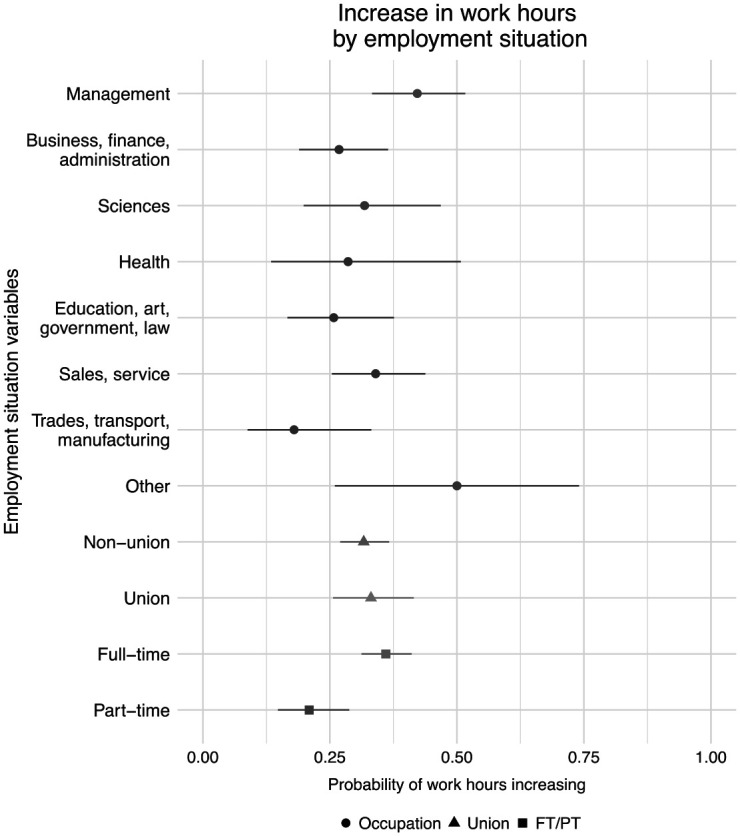
Respondent reports of increased work hours by employment characteristics. *Source.* The 2020 COVID-19 Response Survey of People with Disabilities and Health Conditions, *N* = 490 adults with employment. *Note.* Point estimates and 95 percent confidence intervals.

Indeed, 32.0 percent of respondents reported an increase in work hours since March. Figure 5 shows how the probability of working more hours varied across occupations, union job status, and work hours. Across occupations, work hour increases were more common among respondents in management and sales or service occupations with few differences overall. The main disparities appeared when comparing full-time and part-time workers; 36.0 percent of full-time workers saw an increase in hours compared with 20.9 percent of part-time workers.

Those not working remotely experienced a different change at work. Reagan, who has a second part-time job in a grocery store, compared her two very different work experiences. She felt less affected by changes in her primary government job than her secondary retail job. She remarked about her grocery store job that

There was a huge impact, just because of the social distancing, and because people were coming in in droves when they first announced the schools were closing, to stock up on stuff. In a panic mode. But other than that and the social distancing, all the measures have been put in place, and the constant cleaning and washing at the store. Those are all new, and I don’t think they’re going away, even after—if—COVID ends, but after all this, I don’t think it’s ever going to go away.

Again, we observe how changes in the heavily affected retail sector are affecting workers during the pandemic. Glenn revealed that while he was able to perform his sales duties in a furniture store because of workplace accommodations (he cannot lift or move heavy objects), there were still some struggles at work related to his disability and health condition. With his sales job in jeopardy due to the pandemic, he noted that he simply cannot be moved to another job in the store. He stated, “I can’t jump in and [do] lifting or do bending or long strenuous, strength efforts.” If he did lose his job or get reassigned because of low sales, he told us that “I will be looking for something new. Even if I have to go back into working outside [outdoors] with my health condition, to make more money to keep my family safe, I will.”

In sum, we find that the pandemic has not only affected whether or not people with disabilities and chronic health conditions will enter or exit the labor market, but it has also fundamentally shaped the nature of their jobs. Some have taken on part-time or freelance work that only provides a fraction of what they are used to or expecting. Others saw no interruptions in their income either because they are essential workers or because they are working remotely, both with their own sets of challenges. In addition, we found clear evidence that occupations matter where those in good jobs were less likely to see income disruptions and also expressed a greater sense of financial security.

## Discussion

People with disabilities and chronic illnesses are among the most vulnerable to COVID-19 and the most affected by the very strategies taken to combat the spread of the virus. Although they have voiced their concerns about their higher risk of contracting the virus and about the challenges in subscribing to protective measures, pandemic responses have nonetheless ignored this marginalized group. Our paper illustrates many of the negative impacts of the pandemic on people with disabilities and chronic health conditions, while pointing toward factors that have helped mitigate these negative economic effects. Individuals and households are struggling financially, and those who are unemployed or unable to work due to COVID-19 are among the most affected. Among those who are employed, we find significant variation in the ways work has changed and in levels of financial security by occupation. Finally, we point to the important role of government income supports in helping individuals weather the economic storm brought on by COVID-19.

This study presents findings from one of the few national Canadian surveys regarding the effects of COVID-19 among people with disabilities and chronic health conditions. Due to many of the larger limitations regarding survey research today, including underrepresentation of marginalized groups, quota sampling was extremely useful in examining the immediate effects of health crises on people with disabilities and chronic health conditions. Still, this online survey likely missed people without access to computers or the Internet and individuals with more severe disabilities lacking assistance in accessing the survey. Although quota sampling does not equate to a random sample of the population, our Internet-based sample mirrors the demographic characteristics of members of the population, as discussed in the Supplemental Appendix. Combined with the interview data, our results provide considerable understanding of individuals’ experiences around employment and economic insecurity during a critical time.

Given what we already know about the effects of the COVID-19 pandemic on vulnerable groups, and the worsening situation that came with the second wave in September 2020, more work is needed to uncover specific mechanisms explaining economic disparities in times of crises. For instance, we find important intersections between employment, wealth, and government benefits that should be further investigated as these relate to financial security. Future studies should also take a more systematic look at how people with different disabilities and other health conditions are managing during COVID-19, and how these statuses intersect with race, class, and gender. Finally, drawing from sociological insights on cumulative disadvantage, future studies should address the pandemic’s effects on how pre-employment disadvantage (in education, for example) leads people with disabilities and chronic health conditions into lower paying, less secure employment, further contributing to economic insecurity. This is relevant given that the economic effects of the pandemic extend beyond the labor market and will have long-lasting cumulative effects for years to come.

## Conclusion

COVID-19 is accentuating pre-existing structural disadvantages and inequalities. Although employment levels and income have rebounded for individuals and households who were more advantaged prior to the pandemic, this is not the case for lower income households and less advantaged groups. Many continue to remain insecure with fears of falling off an income cliff. We not only point to the crucial role of employment for economic security but also illustrate that it is not the only factor. This becomes all the more salient during crises that generate exogenous shocks to social, political, and economic systems, whereby individuals cannot necessarily rely on labor market supports alone.

## Supplemental Material

sj-pdf-1-spx-10.1177_07311214211012018 – Supplemental material for Working Differently or Not at All: COVID-19’s Effects on Employment among People with Disabilities and Chronic Health Conditions Supplemental material, sj-pdf-1-spx-10.1177_07311214211012018 for Working Differently or Not at All: COVID-19’s Effects on Employment among People with Disabilities and Chronic Health Conditions by Michelle Lee Maroto, David Pettinicchio and Martin Lukk in Sociological Perspectives

## Notes

1 All respondent names are pseudonyms.

2 Quotas were included for regions during survey collection. We aimed to obtain a sample with 38 percent of responses from Ontario, 23 percent from Québec, 13 percent from British Columbia, 18 percent from the Prairie provinces (Alberta, Manitoba, Saskatchewan), and 7 percent from the Atlantic provinces (Newfoundland and Labrador, New Brunswick, Nova Scotia, and Prince Edward Island) based on 2016 Census population estimates.

3 As is the case with all self-reported survey data, variables are subject to reporting bias.

4 Being unable to work due to COVID-19 includes several scenarios, such as being unable to go to work due to health risks or being employed at a place that was closed due to COVID-19 social distancing/lockdown measures. Respondents elaborated on these scenarios within the qualitative interviews.

5 We classified the majority of provided occupations into one of the six broad categories. We combine other occupations, military occupations, and those without sufficient information to classify in the final category.

6 For comparison, according to the 2017 Canadian Survey on Disability, the employment rate among people with disabilities was 59.4 percent in 2017 (Morris et al. 2018), which indicates a lower employment rate in our sample (47.7 percent) likely due to the pandemic.

7 Estimates for all figures appear in Supplemental Appendix C.
