# Supplementary material for: Working Differently or Not at All: COVID-19’s Effects on Employment among People with Disabilities and Chronic Health Conditions
Source: Sociol Perspect. 2021 May 6;64(5):876–97. doi: 10.1177/07311214211012018 (PMC13038089; doi:10.1177/07311214211012018)
Supplement: sj-pdf-1-spx-10.1177_07311214211012018 – Supplemental material for Working Differently or Not at All: COVID-19’s Effects on Employment among People with Disabilities and Chronic Health Conditions [file sj-pdf-1-spx-10.1177_07311214211012018.pdf]

Appendix A: Survey Data Information and Descriptive Overview

The 2020 COVID-19 Response Survey of People with Disabilities and Health Conditions is a quota-based online survey administered from June 11-22, 2020 with the help of *Qualtrics* (https://www.qualtrics.com), an internet-based survey company, to obtain a sample of people with disabilities and health conditions across 10 Canadian provinces. *Qualtrics* recruits participants from a range of online research panels with which the company partners. These panels consist of pools of people who have been recruited to take regular surveys for academic and corporate researchers. Panelists provide detailed and updated personal information to panel maintainers, including demographic information. *Qualtrics*, as a panel aggregator, matches respondents to surveys based on their profiles and randomly selects them to take surveys that they are likely to quality for, based on a given survey's eligibility criteria.

In the case of our online survey, we provided Qualtrics with eligibility criteria based on age, region, and health and disability status, which it used to recruit participants from appropriate online panels to our survey. Qualtrics takes steps to ensure that a respondent does not take the same survey multiple times or self-select into particular surveys based on the survey topic and incentives. Our survey instrument also included additional screening questions and attention checks to mitigate potential data quality issues. We included location-based sampling quotas to ensure that the proportion of respondents from each province within our final sample matches the proportion of the Canadian population located in each province.

Online samples of the type used in the survey conducted for this study are common in the social sciences and frequently used as an alternative to much costlier traditional survey and sampling methods, such as those based on telephone interviews and random digit dialing.

Context

Data were collected from June 11-22, 2020. During this time many cities and provinces across Canada were beginning to end their "lockdowns" and "open up" their economies. As of June 22, 2020, there were 101,019 confirmed COVID-19 cases in Canada and 8,410 deaths with both cases and deaths trending downward across the country (https://covid19.who.int/region/amro/country/ca).

After seeing its first presumptive cases of COVID-19 at the end of January 2020, Canada began to increase precautions through February and March 2020 with many provinces taking strong measures that included school and business closures, work-from-home mandates, and travel restrictions beginning in mid-March. Although timelines varied across provinces, most began to end these measures in mid-May 2020 working through different phases of "opening up" (https://www.mccarthy.ca/en/insights/articles/covid-19-emergency-measures-tracker; https://theconversation.com/when-will-the-coronavirus-restrictions-end-in-canada-136601).

On April 6, 2020 the federal government established the COVID-19 Emergency Response Act, which aimed to provide a variety of support for individuals and families affected by COVID-19 (https://www.canada.ca/en/department-finance/news/2020/03/introduces-canada-emergency-response-benefit-to-help-workers-and-businesses.html; https://www.canada.ca/en/revenue-

agency/services/benefits/apply-for-cerb-with-cra.html). In particular, the Canada Emergency Response Benefit (CERB) provides up to $2,000 per four-week period for eligible Canadians who are directly affected by COVID-19. This program has been extended through October 2020, and rolled into Employment Insurance.

Respondents

The final survey includes 1,027 respondents age 18 and older who reported having one or more disabilities or health conditions. Disabilities include physical, cognitive, vision, hearing, and emotional limitations. We also included a category related to "other" conditions lasting six months or longer. Health conditions include asthma, cancer, chronic kidney disease, chronic respiratory disease, diabetes, heart disease, hypertension, obesity, and anything associated with being immunocompromised.

To qualify for the survey, respondents had to: (1.) be 18 years or older; (2.) currently reside in a Canadian province; and (3.) indicate that they either (a.) experienced difficulties related to one of the six listed disabilities at least some of the time or (b.) experienced one of the nine listed underlying health conditions. Disability survey questions were based on the Canadian Survey on Disability (Cloutier, Grondin, and Levesque 2018) and guidance provided by the World Health Organization and the Washington Group on Disability Statistics.[1] Health conditions were chosen based on those indicated by the Government of Canada as increasing vulnerability to COVID-19 (https://www.canada.ca/en/public-health/services/publications/diseases-conditions/vulnerable-populations-covid-19.html).

We began with 1,392 respondents. Twenty-nine cases were initially dropped due to poor quality (including gibberish) in their qualitative responses. An additional 51 cases were removed as respondents did not complete the survey. In order to ensure that respondents were carefully reading and answering the survey questions, we included two quality control screening questions that required respondents provide a specific answer. Those who did not correctly answer both quality control questions were screened out of the survey. A final 285 cases were removed because respondents either did not meet the inclusion criteria or failed quality control and attention checks. This resulted in our final sample of 1,027 respondents with no missing data across variables.

Data Quality

The median survey response time was 20 minutes with a mean of 29 minutes. Survey response times ranged from 6 minutes to 15 hours. Longer response times likely reflected instances where respondents began the survey, left it open on their devices, and then went back to complete it later on. Ninety-five percent of respondents completed the survey in under one hour and 80% of respondents completed the survey in less than thirty minutes.

Data were collected via quota-based sampling to ensure that we obtained a representative sample across provinces. Quotas were included only for regions during survey collection. We aimed to obtain a sample with 38% of responses from Ontario, 23% from Quebec, 13% from British

[1] Washington Group on Disability Statistics, https://www.washingtongroup-disability.com

Columbia, 18% from the Prairie provinces (Alberta, Manitoba, Saskatchewan), and 7% from the Atlantic provinces (Newfoundland and Labrador, New Brunswick, Nova Scotia, and Prince Edward Island) based on 2016 Census population estimates.[2]

The survey includes six sets of questions covering disability and chronic health conditions; demographics; COVID-19 responses; income, employment, housing, and economic security; education; and views of government responses to COVID-19. As is the case with most surveys, all variables are self-reported and subject to reporting bias. Justification bias may also be an issue when surveying respondents about disability and employment (Black, Johnston, and Suziedelyte 2017), which is why we made sure to follow guidelines provided by the World Health Organization and the Washington Group on Disability Statistics to assess both the type and severity of disabilities.

Additionally, in order to ensure that respondents were carefully reading and answering the survey questions, we included two quality control screening questions that required respondents provide a specific answer. Those who did not correctly answer both quality control questions were screened out of the survey. We also screened out respondents who answered gibberish within more than one open-ended question.

Descriptives

Table A1 presents descriptive statistics for our survey sample. The mean age for respondents in our sample was 49 years, which is older than the mean age of 41 years for the population in 2016 (Statistics Canada 2020a). This was expected, however, because disabilities and chronic health conditions tend to be more prevalent among older population (CCDSS 2019; Morris et al. 2018).

[Table A1]

The gender make-up of our sample was 53% female, 46% male, and 1% non-binary or other. According to the 2017 CSD, disabilities are more prevalent among women; 56% of people with disabilities were female (Morris et al. 2018, p. 8). Ninety percent of respondents self-identified as heterosexual, 3.5% identified as homosexual (gay or lesbian), 5.0% as bisexual, and 1.3% reported other identities, such as asexual, transgender, and two spirit. Data on sexual orientation are limited, but across several U.S. and Canadian studies 1-2.3% of the population identified as gay or lesbian, 0.7-2.9% as bisexual, and 0.6% as transgender (Waite and Denier 2019). A greater proportion of respondents identified as a sexual minority in our data than as estimated for the larger population. However, additional research shows that rates of disability tend to be higher among lesbian, gay, and bisexual adults (Fredricksen-Goldsen, Kim, and Barkan 2012).

In terms of education, 22.8% of respondents had a high school diploma or less education; 11.1% had some post-secondary education but no diploma; 31.1% completed an apprenticeship, trades, or college certificate; 24.7% had a Bachelor's degree; and 10.3% obtain a degree beyond a Bachelor's, such as a doctorate, master's, or professional degree. Respondents obtained somewhat more education than the population across Canada. Among adults age 25-64 in Canada, 35.3%

---

[2] https://www12.statcan.gc.ca/census-recensement/2016/dp-pd/hlt-fst/pd-pl/Table.cfm?Lang=Eng&T=101&S=50&O=A

had a high school diploma or less (this includes people with some post-secondary education but no degree; 36.3% had an apprenticeship, trades, college certificate, or university certificate below a Bachelor's degree; and 28.5% obtained a Bachelor's degree or higher in 2016 (Statistics Canada 2020b).

Approximately 35.2% of respondents were employed full-time and another 12.6% were employed part-time, 4.4% were unemployed, and 47.9% were not in the labor force, which included homemakers, students, retired persons, and people unable to work due to COVID-19. In this sample, 8.8% of respondents reported that they were unable to work due to COVID-19. According to the 2017 CSD, the employment rate among people with disabilities was 59.4% (Morris et al. 2018), which indicates a lower employment rate in our sample (47.8%). However, percentages are much closer when respondents unable to work due to COVID-19 considered.

Racialized groups are likely underrepresented in this sample. The percentage who identify as Indigenous within our sample (2.3%) was approximately half that of the percentage in the larger population (4.9%) in 2016 (Statistics Canada 2020a). The percentage who identify as non-white in our sample (17.8%) was also lower than the percentage of people who identify as visible minorities in Canada (22.3%). Immigrants and non-citizens were also underrepresented in this sample with 14.4% of respondents indicating they immigrated to Canada compared to 21.9% of the population and 5.5% of respondents indicating that they were not Canadian citizens compared to 7.0% of the population. However, again, it is not clear as to whether these groups are over- or under-represented within groups of people with disabilities and chronic health conditions.

When comparing sample respondents to individuals age 15 and older in Canada, the sample closely mirrored the population in terms of marital status and household size. Within the sample, 53.0% of respondents were married or cohabiting, 31.5% were never married, and 15.5% were formerly married. Across Canada in 2016, 57.4% of individuals age 15 and older were married or in common law relationships, 28.2% were never married, and 14.2% were formerly married (Statistics Canada 2020a).

Disability and chronic health conditions

Table A2 provides further information regarding the prevalence of different disabilities and chronic health conditions among sample participants. Overall, 10.6% of respondents reported a single disability or condition, 35.0% reported 2-3 disabilities or conditions, 32.4% reported 4-5 disabilities or conditions, and 22.0% reported six or more disabilities or conditions.

Disability-related questions allowed respondents to indicate the severity of their disability by reporting whether they sometimes, often, or always experienced any vision, hearing, physical, cognitive, emotional, or other difficulties. Table A2 includes rates of any disability for whether the respondent indicated sometimes, often, or always and rates of more severe disabilities for whether the respondent indicated often or always. In both cases, emotional or other disabilities were the most commonly reported.

Regarding chronic health conditions, diabetes, asthma, hypertension, and obesity were the most commonly chronic health conditions reported by participants. Fewer participants (<6%) reported conditions like cancer, kidney disease, respiratory disease, heart disease, and being immunocompromised.

[Table A2]

Supplementing this overview, Tables A3 and A4 present descriptive statistics for key model variables. Table A3 presents descriptive information for the full sample and Table A4 presents information for employed respondents.

[Table A3]
[Table A4]

Comparison with 2017-18 CCHS data

To further verify the representativeness of our sample, we compare our data to a subsample of the 2017-18 Canadian Community Health Survey (CCHS). Although this survey does not include identical disability and health condition variables, it does provide variables close enough to ours to allow for a comparison of demographics across a subsample of adults age 18+ with reported disabilities or chronic health conditions.

The original 2017-18 CCHS sample included 113,290 cases. Removing missing data for our variables of interest and restricting the sample to adults age 18 and older living in provinces reduced our sample size to 94,797 cases. We then restricted this sample to individuals who reported one or more disabilities or chronic health conditions, which resulted in a final sample size of 60,082 cases.

Within this sample a respondent is considered to have a disability if they indicated that they had some difficulty, a lot of difficulty, or inability (cannot do at all) in regard to seeing (even with glasses), hearing (even with a hearing aid), walking or climbing stairs, remembering or concentrating, self-care, or communicating. In order to include emotional disabilities, we also counted individuals who reported that they had a major mood or anxiety disorder.

We also included individuals with the following chronic health conditions: asthma, respiratory conditions, hypertension, heart diseases, diabetes, and cancer. Although we also wanted to include obesity in this analysis, we omitted this variable due to the amount of missing data with it. The survey also did not include questions regarding being immunocompromised.

Table A5 presents information regarding the prevalence of disability and chronic health conditions in the full CCHS sample. In the larger sample, 43.7% of respondents reported any disability (having at least some difficulty across designated areas) and 18.1% reported a more severe disability (having a lot of difficulty or cannot do at all). Additionally, 30.9% of respondents reported having one of six designated chronic health conditions.

[Table A5]

Table A6 presents descriptive statistics for demographic variables in the CCHS subset of people with disabilities or chronic health conditions. Comparing these to data from the 2020 COVID-19 Disability Survey, the CCHS sample is slightly older with a mean age of 51.6 years compared to 49.0 years in the COVID-19 Disability Survey. Gender composition is similar across surveys with a slightly greater proportion of men in both. Notably, the CCHS did not permit respondents to report a gender other than male or female. We also do not include a comparison based on sexual identity due to the larger percentage of missing data on this variable in the CCHS.

Similar to earlier comparisons with national population data, a comparison with the CCHS again shows that immigrants and Indigenous persons were underrepresented within the COVID-19 Disability Survey sample. It also shows that our sample has attained somewhat higher levels of education with 66.1% of the COVID-19 Disability Survey obtaining a PSE certificate or higher compared to 59.1% of CCHS respondents. Employment rates were closer across surveys. In the COVID-19 Disability Survey, 47.7% of respondents were employed at the time of the survey compared to 50.0% of CCHS respondents. As expected during the pandemic, the unemployment rate among COVID-19 Disability Survey respondents was higher.

The CCHS marital status variable categories differ from those used in the COVID-19 Disability Survey, but it appears as those marriage rates were lower in the COVID-19 Disability Survey. Within the COVID-19 Disability Survey 39.5% of respondents were married, 13.4% were cohabiting, 15.5% were formerly married, and 31.5% were never married. Within the CCHS, 50.0% of respondents were married, 11.8% were in common law unions, 16.1% were formerly married, and 22.1% were single.

[Table A6]

Finally, Table A7 presents information regarding the prevalence of different types of disabilities and chronic health conditions in the CCHS subsample. This table shows that most specific disabilities were more prevalent across respondents in the COVID-19 Disability Survey. A greater percentage of COVID-19 Disability Survey respondents were also more likely to report most conditions, except for hypertension and heart disease, which were more prevalent in the CCHS subsample.

It is important to note that the ways in which questions were phrased and the options given to respondents differ across surveys. For instance, COVID-19 Disability Survey respondents were given options of "no," "sometimes," "always," and "never." CCHS respondents were given options of "no difficulty," "some difficulty," "a lot of difficulty," and "cannot do at all / unable to do."

[Table A7]

Overall, this comparison between the 2020 COVID-19 Disability Survey and the 2017-18 CCHS subsample of people with disabilities and chronic health conditions shows both similarities and differences across survey respondents. Although age and gender composition are similar across

survey respondents, differences do appear in regard to race and education. It is important to note that the disabilities and conditions included across surveys are not identical, however, a factor that could also affect the composition of survey respondents.

Table A1 Descriptive Overview of Data

	Sample Frequency	Proportion or Mean
Age (mean years)	--	48.999
Gender		
Male	472	0.460
Female	544	0.530
Non-binary or other	11	0.011
Sexual identity		
Heterosexual	927	0.903
Homosexual	36	0.035
Bisexual	51	0.050
Other	13	0.013
Member of a racialized minority group	184	0.179
Immigrant	148	0.144
Indigenous	24	0.023
First language other than English or French	50	0.049
Employment status (reduced variable)		
Employed	490	0.477
Unemployed	45	0.044
NILF (homemaker, retired, in school)	402	0.391
Unable to work due to COVID-19	90	0.088
Education		
HS or less	234	0.228
Some PSE, no degree	114	0.111
Apprenticeship, trades, or college certificate	319	0.311
Bachelor's degree	254	0.247
Advanced degree	106	0.103
Marital status		
Never married	324	0.315
Cohabiting	138	0.134
Married	406	0.395
Formerly married	159	0.155
Any children	246	0.240
Number of adults in household		
Single adult (self)	329	0.320
Two adults	484	0.471
Three adults	124	0.121
Four adults	65	0.063
Five or more adults	25	0.024
Province (reduced)		
Ontario	399	0.389
Quebec	230	0.224
BC	134	0.130
Prairie provinces	199	0.194
Atlantic provinces	65	0.063

SOURCE: 2020 COVID-19 Response Survey of People with Disabilities and Health Conditions, N = 1027 adults
NOTES: Estimates refer to sample data. Estimates provided as proportions unless otherwise specified.

Table A2 Descriptive Statistics for Disability and Chronic Health Condition Variables

	Sample Frequency	Proportion or Mean
Number of disabilities or chronic health conditions		
One	109	0.106
Two or three	359	0.350
Four or five	333	0.324
Six or more	226	0.220
Disability type (Any)		
Vision	558	0.543
Hearing	270	0.263
Physical	433	0.422
Cognitive	502	0.489
Emotional	565	0.550
Other	591	0.575
Disability type (Always or Often)		
Vision	120	0.117
Hearing	54	0.053
Physical	131	0.128
Cognitive	128	0.125
Emotional	269	0.262
Other	426	0.415
Chronic health condition		
Asthma	249	0.242
Cancer	42	0.041
Chronic kidney disease	16	0.016
Chronic respiratory or lung disease	54	0.053
Diabetes	262	0.255
Hypertension	221	0.215
Obesity	163	0.159
Immunocompromised	51	0.050
Heart disease	56	0.055

SOURCE: 2020 COVID-19 Response Survey of People with Disabilities and Health Conditions, N = 1027 adults

NOTES: Estimates refer to sample data. Estimates provided as proportions unless otherwise specified.

Table A3 Descriptive Statistics, Key Variables, All Respondents

	Frequency	Percentage
Financial situation worse than previous year	406	39.53
COVID-19-specific effects on financial outcomes	485	47.22
Detailed employment status		
Employed full-time	361	35.15
Employed part-time	129	12.56
Not working, looking for work	45	4.38
Not working, homemaker	28	2.73
Not working, in school	33	3.21
Not working, retired	256	24.93
Not working, unable to work due to COVID-19	90	8.76
Not working, unable to work for other reasons	85	8.28
Applied for CERB		
Have not applied for CERB and have no plans to	753	73.32
Have not applied for CERB but plan to	53	5.16
Have already applied for CERB	221	21.52

SOURCE: 2020 COVID-19 Response Survey of People with Disabilities and Health Conditions, N = 1027 adults

Table A4 Descriptive Statistics, Key Variables, Employed Respondents

	Frequency	Percentage
Expect to lose job due to COVID-19 in next year	249	50.82
Work from home due to COVID-19	276	56.33
Taken on more work due to COVID-19	157	32.04
Major occupation for current job		
Management occupations	109	22.24
Business, finance and administration occupations	97	19.80
Natural and applied sciences and related occupations	44	8.98
Health occupations	20	4.08
Occupations in education, law, community, government, arts, culture	67	13.67
Sales and service occupations	100	20.41
Occupations in trades, transport and equipment, natural resources, manufacturing, and utilities	39	7.96
Other, military, NEC	14	2.86
Part-time employment	129	26.33
Union membership	133	27.14

SOURCE: 2020 COVID-19 Response Survey of People with Disabilities and Health Conditions, N = 490 persons with employment

Table A5 Descriptive Statistics for Full Sample, 2017-18 CCHS

	Sample Frequency	Proportion or Mean		
		Estimate	95% Confidence Interval	
			Lower	Upper
Disability (Any)	47223	0.437	0.432	0.443
Disability (More severe)	19941	0.181	0.177	0.185
Health condition	35458	0.309	0.304	0.314

SOURCE: 2017-18 CCHS, N = 94,794 adults age 18+ living in provinces

NOTES: Estimates include survey provided sample weights

Table A6 Descriptives Statistics for Demographic Variables, 2017-18 CCHS Subsample

	Sample Frequency	Proportion or Mean		
		Estimate	95% Confidence Interval	
			Lower	Upper
Age (mean years)	--	51.601	51.341	51.861
Gender				
Male	26988	0.480	0.472	0.487
Female	33094	0.520	0.513	0.528
Immigrant	9056	0.241	0.234	0.248
Indigenous	3164	0.042	0.040	0.045
Main activity				
Paid work	24377	0.500	0.493	0.507
Unemployed	1570	0.028	0.026	0.030
NILF	34135	0.472	0.465	0.479
Education				
Less than HS	11462	0.151	0.146	0.155
HS graduate	15002	0.258	0.252	0.265
Post-secondary education degree or higher	33618	0.591	0.584	0.598
Marital status				
Married	25942	0.500	0.493	0.507
Common law	5660	0.118	0.114	0.123
Formerly married	16134	0.161	0.156	0.165
Single	12346	0.221	0.215	0.227
Household size				
Single person (self)	21015	0.187	0.182	0.191
Two people	24636	0.397	0.390	0.403
Three people	6698	0.173	0.167	0.178
Four people	5022	0.148	0.142	0.154
Five or more people	2711	0.096	0.091	0.102
Living situation				
Unattached individual living alone	21015	0.187	0.182	0.191
Unattached individual living with others	1810	0.044	0.041	0.047
Individual living with spouse/partner	20602	0.323	0.317	0.329
Parent living with spouse/partner and child(ren)	8590	0.229	0.223	0.236
Single parent living with children	3184	0.051	0.048	0.054
Child living with a single parent with or without siblings	1034	0.028	0.025	0.031
Child living with two parents with or without siblings	1617	0.056	0.052	0.060
Other	2230	0.082	0.077	0.088
Province (reduced)				
Ontario	18208	0.383	0.375	0.390
Quebec	12643	0.226	0.220	0.231
BC	8208	0.134	0.129	0.138
Prairie provinces	12953	0.183	0.178	0.188
Atlantic provinces	8070	0.075	0.072	0.078

SOURCE: 2017-18 CCHS, N = 60,082 adults age 18+ with reported disabilities or chronic health conditions living in provinces
NOTES: Estimates include survey-provided sample weights Estimates provided as proportions unless otherwise specified.

Table A7 Descriptive Statistics for Disability and Chronic Health Condition Variables, 2017-18 CCHS Subsample

	Sample Frequency	Proportion or Mean		
		Estimate	95% Confidence Interval	
			Lower	Upper
Disability type (Any)				
Vision	14723	0.243	0.237	0.249
Hearing	14642	0.214	0.208	0.219
Physical	18292	0.253	0.247	0.259
Cognitive	18585	0.308	0.302	0.315
Self-care	3677	0.054	0.051	0.057
Communication	4210	0.072	0.068	0.075
Emotional	13957	0.236	0.230	0.243
Disability type (More severe)				
Vision	1247	0.018	0.016	0.020
Hearing	1760	0.024	0.022	0.026
Physical	5327	0.067	0.064	0.070
Cognitive	2062	0.035	0.033	0.038
Self-care	793	0.012	0.010	0.013
Communication	459	0.008	0.006	0.009
Chronic health condition				
Asthma	7975	0.140	0.135	0.144
Cancer	2097	0.029	0.027	0.032
Chronic respiratory or lung disease	4297	0.050	0.048	0.053
Diabetes	8968	0.133	0.128	0.137
Hypertension	22108	0.325	0.319	0.332
Heart disease	6390	0.084	0.080	0.087

SOURCE: 2017-18 CCHS, N = 60,082 adults age 18+ with reported disabilities or chronic health conditions living in provinces

NOTES: Estimates include survey-provided sample weights Estimates provided as proportions unless otherwise specified.

Appendix B: Interview Data Information and Methods

At the end of each survey, respondents were given the option of providing emails or phone numbers if they wished to be contacted for a follow-up interview. Five hundred and six respondents requested to be contacted for follow-up interviews. To fairly determine who to invite for an interview, we first took a random sample of 100 respondents, narrowed this list down to ensure that people with different disabilities, health conditions, and other characteristics (i.e., age, gender, race) were represented in our interview sample. We then contacted people on this list via email until reaching 50 interview participants.

Interviews took place between August and November 2020. Research assistants interviewed respondents by telephone. Interviews ranged 12 to 60 minutes in length with a mean length of 33.9 minutes and a median length of 33.4. All interviews were audio recorded and then transcribed using the transcription service *Rev*. Once transcribed, research assistants then reviewed each transcript to check for any errors or omissions.

After gathering and transcribing the interview data, the research team qualitatively coded all transcripts to identify major themes among respondents' in-depth interview responses. This process involved applying codes associated with the project's central research questions, as well as developing codes based on emergent themes.

Interview analysis and coding occurred through an iterative process. The analysis began with developing a set of codes using a combination of both inductive and deductive approaches. Team members first collaboratively identified a set of key analytic themes associated with COVID-19's effects on employment among people with disabilities and chronic health conditions. These initial deductive themes fell under the broad categories of health and disability, employment and finances, social life, and public policy and included specific themes dealing with topics like the cost of living, working from home, and applying for government aid programs (e.g., CERB). Initial deductive codes reflected our expectations based on the literature and our quantitative survey and partially determined by the interview design itself.

In addition to generating deductive themes, we also generated a preliminary set of inductive themes prior to analysis of the complete interview data set. All team members independently reviewed a predetermined subset of interviews and each generated a set of inductive codes based on patterns of responses in the interviews. These included many specific themes that emerged from the data, such as "changing spending habits," "heightened sense of worry," and "loneliness." Team members met virtually after independently coding the initial subset of interviews and discussed these themes. Team members' themes were compared and reconciled, to generate a coding scheme that ultimately drew on both deductive and inductive themes.

This collaboratively developed draft coding scheme was then used to analyze another small subset of interviews, to ensure it could be used consistently between coders and that it sufficiently covered the interview subject matter. This stage served as a pre-test of the coding scheme. At this stage, final adjustments were made to arrive at a final coding scheme. Two coders analyzed the full data set of 50 interviews using these codes, which covered topics central

to the study's research questions. Interviews were thematically coded using *Dedoose*, a web-based qualitative data analysis software program.

Interview Sample Description

Table B1 presents descriptive statistics for key demographic variables across three samples – the full survey sample (N = 1,027), the sample of respondents who indicated that they would be willing to be interviewed (N = 506), and the sample of interview participants (N = 50).

[Table B1]

As shown in Table B1, the interview sample is slightly older than the survey sample with a mean of 52.4 years compared to 49.0 years. Women were overrepresented in all samples with the greatest overrepresentation in the interview sample. Approximately 58% of this sample identified as female and 42% identified as male. No interview participants identified as non-binary or in other gender categories. The majority of participants in all samples identified as heterosexual. In the interview sample, 86% of participants identified as heterosexual, 4.0% identified as homosexual, gay, or lesbian, and 10.0% identified as bisexual. As noted, members of racial minority groups were underrepresented across samples with 18.0% of interview respondents indicating membership in a racial minority group, 4.0% identifying as Indigenous, and 14.0% reporting having immigrated to Canada.

Employment rates were similar across samples with slightly more interview participants being employed than survey participants. Most interview participants were employed (50%) or not in the labor force (42%). Only one interview participant was unemployed and three were unable to work due to COVID-19. Interview participants also reported higher levels of education with 42% having Bachelor's or advanced degrees compared to 35% of survey participants.

Marital and parental statuses were similar across samples. In the interview sample, 32% of participants were never married, 12% were cohabiting, 42% were currently married, and 14% were formerly married. Eleven interview participants (22%) had children. Fewer interview participants reported living alone with 26% living alone compared to 32% in the full survey sample. Finally, provincial representation was more skewed toward Ontario in the interview sample. Thirty interview participants (60%) lived in Ontario, two live in Québec, eight lived in BC, eight lived in the Prairie provinces, and two lived in the Atlantic provinces.

Table B2 presents descriptive statistics for disability and chronic health condition variables across same three samples – the full survey sample (N = 1,027), the sample of respondents who indicated that they would be willing to be interviewed (N = 506), and the sample of interview participants (N = 50).

[Table B2]

Like the survey respondents, most interview participants reported having multiple disabilities or chronic health conditions. Twenty-one (42%) reported having 2-3 disabilities or conditions, 13 reported 4-5, and 12 reported 6 or more. Vision, emotional, and other (not specified) disabilities

were the most common. Among health conditions, hypertension, asthma, and obesity were most often reported.

For reference, Table B3 includes information on interview respondents with the reported gender, ages, disability or chronic health condition, employment status, and marital status.

[Table B3]

Table B1 Descriptives statistics for demographic variables across samples

	Full sample		Willing to be interviewed		Interviewed	
	Frequency	Proportion or Mean	Frequency	Proportion or Mean	Frequency	Proportion or Mean
Age (mean years)	--	48.999	--	51.225	--	52.380
Gender						
Male	472	0.460	237	0.468	21	0.420
Female	544	0.530	264	0.522	29	0.580
Non-binary or other	11	0.011	5	0.010	0	--
Sexual identity						
Heterosexual	927	0.903	453	0.895	43	0.860
Homosexual	36	0.035	20	0.040	2	0.040
Bisexual	51	0.050	25	0.049	5	0.100
Other	13	0.013	8	0.016	0	--
Member of a racialized minority group	184	0.179	81	0.160	9	0.180
Immigrant	148	0.144	68	0.134	7	0.140
Indigenous	24	0.023	12	0.024	2	0.040
First language other than English or French	50	0.049	24	0.047	0	--
Employment status (reduced variable)						
Employed	490	0.477	226	0.447	25	0.500
Unemployed	45	0.044	19	0.038	1	0.020
NILF (homemaker, retired, in school)	402	0.391	211	0.417	21	0.420
Unable to work due to COVID-19	90	0.088	50	0.099	3	0.060
Education						
HS or less	234	0.228	92	0.182	8	0.160
Some PSE, no degree	114	0.111	54	0.107	3	0.060
Apprenticeship, trades, or college certificate	319	0.311	163	0.322	18	0.360
Bachelor's degree	254	0.247	138	0.273	17	0.340
Advanced degree	106	0.103	59	0.117	4	0.080
Marital status						
Never married	324	0.315	159	0.314	16	0.320
Cohabiting	138	0.134	62	0.123	6	0.120
Married	406	0.395	195	0.385	21	0.420
Formerly married	159	0.155	90	0.178	7	0.140
Any children	246	0.240	106	0.209	11	0.220
Number of adults in household						
Single adult (self)	329	0.320	169	0.334	13	0.260
Two adults	484	0.471	236	0.466	28	0.560
Three adults	124	0.121	61	0.121	5	0.100
Four adults	65	0.063	29	0.057	2	0.040
Five or more adults	25	0.024	11	0.022	2	0.040
Province (reduced)						
Ontario	399	0.389	219	0.433	30	0.600
Quebec	230	0.224	99	0.196	2	0.040
BC	134	0.130	64	0.126	8	0.160
Prairie provinces	199	0.194	97	0.192	8	0.160
Atlantic provinces	65	0.063	27	0.053	2	0.040
N	1027		506		50	

SOURCE: 2020 COVID-19 Response Survey of People with Disabilities and Health Conditions and Interview Panel

NOTES: Estimates refer to sample data. Estimates provided as proportions unless otherwise specified.

Table B2 Descriptives statistics for disability and chronic health condition variables across samples

	Full sample		Willing to be interviewed		Interviewed	
	Frequency	Proportion or Mean	Frequency	Proportion or Mean	Frequency	Proportion or Mean
Number of disabilities or chronic health conditions						
One	109	0.106	48	0.095	4	0.080
Two or three	359	0.350	175	0.346	21	0.420
Four or five	333	0.324	152	0.300	13	0.260
Six or more	226	0.220	131	0.259	12	0.240
Disability type (Any)						
Vision	558	0.543	268	0.530	25	0.500
Hearing	270	0.263	134	0.265	15	0.300
Physical	433	0.422	229	0.453	21	0.420
Cognitive	502	0.489	241	0.476	14	0.280
Emotional	565	0.550	287	0.567	25	0.500
Other	591	0.575	310	0.613	34	0.680
Disability type (Always or Often)						
Vision	120	0.117	57	0.113	9	0.180
Hearing	54	0.053	26	0.051	2	0.040
Physical	131	0.128	73	0.144	4	0.080
Cognitive	128	0.125	59	0.117	2	0.040
Emotional	269	0.262	130	0.257	7	0.140
Other	426	0.415	224	0.443	24	0.480
Chronic health condition						
Asthma	249	0.242	124	0.245	12	0.240
Cancer	42	0.041	25	0.049	1	0.020
Chronic kidney disease	16	0.016	9	0.018	2	0.040
Chronic respiratory or lung disease	54	0.053	30	0.059	0	--
Diabetes	262	0.255	135	0.267	10	0.200
Hypertension	221	0.215	119	0.235	18	0.360
Obesity	163	0.159	96	0.190	14	0.280
Immunocompromised	51	0.050	26	0.051	4	0.080
Heart disease	56	0.055	32	0.063	1	0.020
N	1027		506		50	

SOURCE: 2020 COVID-19 Response Survey of People with Disabilities and Health Conditions and Interview Panel

NOTES: Estimates refer to sample data. Estimates provided as proportions unless otherwise specified.

Table B3 Interview data description table

Respondent	Gender	Age	Disability or Health Condition	Employment Status	Marital Status
Evan	Male	36	Asthma	Employed full-time	Cohabiting
Jean-Marc	Male	29	Emotional disability	Employed full-time	Never married
Lina	Female	36	Cognitive, emotional, other disability / Asthma	Not working, homemaker	Never married
Donald	Male	53	Other disability / Hypertension	Employed full-time	Never married
Maryam	Female	35	Emotional, other disability / Obesity	Employed full-time	Never married
Nicki	Female	50	Vision, other disability / Asthma, kidney condition, diabetes, hypertension, obesity	Not working, homemaker	Married
Antoine	Male	72	Hearing, physical, cognitive, emotional, other disability / Diabetes, hypertension	Employed part-time	Married
Esther	Female	60	Vision, hearing, physical, emotional, other disability / Hypertension	Not working, looking for work	Cohabiting
Sydney	Female	71	Other disability / Asthma, immunosuppressed	Not working, retired	Formerly married
Glenn	Male	46	Vision, hearing, physical, cognitive, emotional, other disability / Diabetes	Employed full-time	Married
Dillan	Male	37	Vision, physical, emotional, other disability / Hypertension, immunosuppressed	Employed full-time	Married
Natalie	Female	82	Physical, other disability / Hypertension	Not working, retired	Formerly married
Pat	Female	58	Emotional disability / Obesity	Not working, retired	Married
Reagan	Female	61	Vision, hearing, physical, cognitive disability / Asthma, diabetes, hypertension, obesity	Employed full-time	Never married
Dawn	Female	52	Vision, cognitive, emotional disability	Not working, homemaker	Formerly married
Lynette	Female	61	Emotional, other disability	Not working, homemaker	Married
Drew	Male	49	Vision, emotional, other disability	Not working, unable to work due to COVID-19	Never married
Bailey	Female	36	Vision, hearing, physical, cognitive, emotional, other disability / Immunosuppressed	Not working, unable to work due to COVID-19	Never married
Jasmine	Female	47	Vision, hearing disability	Employed full-time	Married
Cheyenne	Female	27	Physical, other disability / Diabetes	Employed full-time	Never married
Daniel	Male	66	Vision, hearing, physical, other disability / Diabetes, hypertension, heart condition	Not working, retired	Formerly married
Su-anne	Female	50	Vision, physical, cognitive, emotional, other disability / Obesity	Not working, unable to work for other reasons	Formerly married
Dina	Female	57	Vision, hearing, physical, cognitive, other disability / Immunosuppressed	Not working, unable to work for other reasons	Cohabiting
Ramona	Female	28	Vision, hearing, cognitive, emotional, other disability	Employed full-time	Never married

Respondent	Gender	Age	Disability or Health Condition	Employment Status	Marital Status
Doreen	Female	63	Vision, hearing, emotional disability / Asthma	Not working, retired	Married
Robert	Male	60	Vision, physical disability	Not working, retired	Married
Dick	Male	78	Hearing, physical, other disability / Diabetes, hypertension	Not working, retired	Married
Wes	Male	72	Vision, other disability / Cancer, hypertension	Not working, retired	Married
Dora	Female	76	Physical, other disability / Asthma, hypertension	Not working, unable to work due to COVID-19	Formerly married
Gene	Male	59	Other disability / Hypertension	Employed full-time	Married
Aayan	Female	31	Vision, emotional, other disability	Employed full-time	Never married
Courtney	Female	36	Vision, hearing disability / Diabetes, hypertension	Employed full-time	Married
Darren	Male	64	Other disability / Diabetes, hypertension	Employed full-time	Married
Angelica	Female	67	Physical, other disability / Kidney condition, hypertension, obesity	Not working, retired	Formerly married
DJ	Male	53	Vision, cognitive disability / Asthma	Employed full-time	Married
Timothy	Male	42	Physical, emotional, other disability	Employed full-time	Married
Seth	Male	38	Asthma	Employed full-time	Married
Eve	Female	66	Emotional disability / Obesity	Employed part-time	Cohabiting
Kelsey	Female	35	Vision, hearing, cognitive, emotional, other disability / Diabetes	Employed full-time	Never married
Norman	Male	60	Physical, emotional disability / Hypertension, obesity	Employed full-time	Married
Margo	Female	71	Vision, hearing, physical, other disability /Obesity	Not working, retired	Never married
Dorothy	Female	61	Vision, physical, cognitive, emotional, other disability / Diabetes, obesity	Not working, homemaker	Married
Kurtis	Male	40	Emotional disability / Obesity	Employed full-time	Never married
Allison	Female	47	Other disability / Asthma, obesity	Employed full-time	Cohabiting
Eugenie	Female	62	Other disability	Not working, unable to work for other reasons	Married
Jacynthe	Female	35	Vision, cognitive, emotional disability	Not working, homemaker	Never married
Slater	Male	33	Vision, physical, emotional, other disability	Not working, unable to work for other reasons	Never married
Beck-Ann	Female	73	Hearing, physical disability / Hypertension	Not working, retired	Married
Owen	Male	40	Vision, cognitive, emotional, other disability / Asthma	Employed full-time	Cohabiting
Langston	Male	58	Other disability / Asthma, hypertension, obesity	Employed full-time	Never married

SOURCE: 2020 COVID-19 Response Interviews with People with Disabilities and Health Conditions, N = 50 adults

Appendix C: Model Results for Figures

The following set of tables (Tables C1-C5) provides model results and coefficients for each of the sets of predicted probabilities that appear in figures (Figures 1-5) in the manuscript.

Table C1 Bivariate Logit Models Predicting Worsening Financial Condition by Employment Status

	b	SE	AME	SE
Intercept	-.648***	(.111)		
Employment status (Ref: Employed, full-time)				
Employed part-time	-.045	(.217)	-.010	(.048)
Not working, looking for work	1.443***	(.341)	.345***	(.073)
Not working, homemaker	-.099	(.420)	-.022	(.092)
Not working, in school	.830*	(.367)	.202*	(.090)
Not working, retired	.036	(.172)	.008	(.039)
Not working, unable to work due to COVID-19	1.007***	(.241)	.245***	(.058)
Not working, unable to work for other reasons	.435	(.245)	.104	(.059)
Pseudo R-squared	.031			

*** p<.001, ** p<.01, * p<.05

SOURCE: 2020 COVID-19 Response Survey of People with Disabilities and Health Conditions, N = 1027 adults

NOTES: Bivariate logistic regression models predicting reports of a worsening financial status. Estimates (b) and standard errors (SE), and average marginal effects (AME) and standard errors (SE). Basis for predicted probabilities in Figure 1.

Table C2 Bivariate Logit Models Predicting COVID-19 Negative Financial Effects by Employment Status

	b	SE	AME	SE
Intercept	-.116	(.105)		
Employment status (Ref: Employed, full-time)				
Employed part-time	.070	(.205)	.018	(.051)
Not working, looking for work	1.017**	(.345)	.240***	(.073)
Not working, homemaker	-.471	(.408)	-.114	(.094)
Not working, in school	.299	(.365)	.075	(.091)
Not working, retired	-.783***	(.174)	-.182***	(.039)
Not working, unable to work due to COVID-19	1.128***	(.261)	.262***	(.054)
Not working, unable to work for other reasons	.571*	(.246)	.141*	(.059)
Pseudo R-squared	.057			

*** p<.001, ** p<.01, * p<.05

SOURCE: 2020 COVID-19 Response Survey of People with Disabilities and Health Conditions, N = 1027 adults

NOTES: Bivariate logistic regression models predicting reports of negative financial effects of COVID-19 by employment status. Estimates (b) and standard errors (SE), and average marginal effects (AME) and standard errors (SE). Basis for predicted probabilities in Figure 2.

Table C3 Bivariate Logit Models Predicting Concerns about Job Loss by Work Situation Variables

	b	SE	AME	SE
**Occupation model**				
Intercept	.277	(.193)		
Major occupation (Ref: Management occupations)				
Business, finance and administration occupations	-.422	(.281)	-.105	(.069)
Natural and applied sciences and related occupations	-.186	(.358)	-.046	(.089)
Health occupations	-1.724**	(.588)	-.378***	(.098)
Occupations in education, law, community, government, arts, culture	.028	(.315)	.007	(.077)
Sales and service occupations	-.357	(.278)	-.089	(.069)
Occupations in trades, transport and equipment, natural resources, manufacturing, and utilities	-.226	(.374)	-.056	(.093)
Other, military, NEC	.311	(.590)	.074	(.137)
Pseudo R-Squared	.021			
**Union model**				
Intercept	.219*	(.107)		
Non-union worker	-.694***	(.208)	-.171***	(.050)
Pseudo R-Squared	.017			
**Full-time/part-time status model**				
Intercept	-.017	(.105)		
Part-time worker	.188	(.206)	.047	(.051)
Pseudo R-Squared	.001			

*** p<.001, ** p<.01, * p<.05

SOURCE: 2020 COVID-19 Response Survey of People with Disabilities and Health Conditions, N = 490 persons with employment

NOTES: Bivariate logistic regression models predicting concerns about job loss by employment characteristics. Estimates (b) and standard errors (SE), and average marginal effects (AME) and standard errors (SE). Basis for predicted probabilities in Figure 3.

Table C4 Bivariate Logit Models Predicting Transition to Work from Home by Work Situation Variables

	b	SE	AME	SE
**Occupation model**				
Intercept	1.015***	(.217)		
Major occupation (Ref: Management occupations)				
Business, finance and administration occupations	-.352	(.305)	-.074	(.064)
Natural and applied sciences and related occupations	.343	(.432)	.062	(.074)
Health occupations	-2.462***	(.597)	-.544***	(.096)
Occupations in education, law, community, government, arts, culture	-.182	(.345)	-.037	(.071)
Sales and service occupations	-1.678***	(.303)	-.394***	(.064)
Occupations in trades, transport and equipment, natural resources, manufacturing, and utilities	-2.535***	(.470)	-.555***	(.075)
Other, military, NEC	-1.302*	(.582)	-.305*	(.139)
Pseudo R-Squared	.135			
**Union model**				
Intercept	.357***	(.108)		
Non-union worker	-.372	(.204)	-.092	(.051)
Pseudo R-Squared	.005			
**Full-time/part-time status model**				
Intercept	.433***	(.108)		
Part-time worker	-.667**	(.208)	-.165**	(.051)
Pseudo R-Squared	.016			

*** p<.001, ** p<.01, * p<.05

SOURCE: 2020 COVID-19 Response Survey of People with Disabilities and Health Conditions, N = 490 persons with employment

NOTES: Bivariate logistic regression models predicting transition to work from home by employment characteristics. Estimates (b) and standard errors (SE), and average marginal effects (AME) and standard errors (SE). Basis for predicted probabilities in Figure 4.

Table C5 Bivariate Logit Models Predicting Increased Work Hours by Work Situation Variables

	b	SE	AME	SE
**Occupation model**				
Intercept	-.315	(.194)		
Major occupation (Ref: Management occupations)				
Business, finance and administration occupations	-.690*	(.300)	-.154*	(.065)
Natural and applied sciences and related occupations	-.448	(.377)	-.104	(.085)
Health occupations	-.602	(.521)	-.136	(.109)
Occupations in education, law, community, government, arts, culture	-.744*	(.342)	-.164*	(.072)
Sales and service occupations	-.349	(.287)	-.082	(.067)
Occupations in trades, transport and equipment, natural resources, manufacturing, and utilities	-1.205**	(.460)	-.243**	(.078)
Other, military, NEC	.315	(.569)	.078	(.142)
Pseudo R-Squared	.022			
**Union model**				
Intercept	-.770***	(.114)		
Non-union worker	.065	(.217)	.014	(.048)
Pseudo R-Squared	.000			
**Full-time/part-time status model**				
Intercept	-.575***	(.110)		
Part-time worker	-.754**	(.243)	-.151***	(.044)
Pseudo R-Squared	.017			

*** p<.001, ** p<.01, * p<.05

SOURCE: 2020 COVID-19 Response Survey of People with Disabilities and Health Conditions, N = 490 persons with employment

NOTES: Bivariate logistic regression models predicting increased work hours by employment characteristics. Estimates (b) and standard errors (SE), and average marginal effects (AME) and standard errors (SE). Basis for predicted probabilities in Figure 5.

Appendix D: Employment Rate Comparisons with the Full Population

Appendix D provides comparisons of employment rates across the Canadian population.

Analysis of June 2020 Labour Force Survey

The Labour Force Survey (LFS) is a long-running monthly survey conducted by Statistics Canada. Although it does not include information on disability, it covers employment and unemployment statistics, as well as variation by industry, occupation, sector, and demographics.

We use data from the 2020 Labour Force Survey to provide a comparison with employment rates for the population. We restrict these data to adults age 17 and older, resulting in a sample size of 85,470 cases. Table D1 presents survey-weighted estimates for labor force status and hours worked per week at all jobs.

[Table D1]

As shown in Table D1, 51% of Canadian adults were employed and at work in June 2020, 7.0% were employed but absent from work, 7.6% were unemployed, and 34.4% were not in the labor force. As expected, these employment rates were higher than for our survey participants (Table A1) where 47.7% were employed, 4.4% were unemployed, and 47.9% were not in the labor force.

Analysis of May 2020 Canadian Perspective Survey Series 2: Monitoring the Effects of COVID-19

In response to COVID-19, Statistics Canada launched a series of short online surveys, the Canadian Perspective Survey Series, to capture the changing situation. These surveys are based on Statistics Canada's probability panel, comprising a subset of participants from the Labour Force Survey.

We analyze data from the second of these surveys, Canadian Perspective Survey Series 2: Monitoring the Effects of COVID-19 (CPSS2), which was administered from May 4, 2020 until May 10, 2020. After removing missing data on variables of interest, we retained a sample of 4,551 respondents age 15 and older. Due to age variable groupings, we were not able to limit the sample to adults. Table D2 presents survey-weighted estimates for employment and COVID-19-related variables.

[Table D2]

As shown in Table D2, 46.3% of respondents were employed and at work in May 2020, 2.6% were employed but absent from work for reasons other than COVID-19, 6.8% were employed but absent from work due to COVID-19, and 44.3% were employed. Among employed workers, 16.0% were concerned they might lose their jobs in the next four weeks. Finally, 46.2% indicated that COVID-19 had affected their ability to meet their financial needs in some way.

Table D1 Descriptives Statistics for Employment Variables, June 2020 LFS

	Sample Frequency	Proportion or Mean		
		Estimate	95% Confidence Interval	
			Lower	Upper
Labour force status				
Employed, at work	42741	0.510	0.505	0.514
Employed, absent from work	5561	0.070	0.068	0.073
Unemployed	5456	0.076	0.073	0.079
Not in labour force	31712	0.344	0.340	0.349
Actual hours worked per week at all jobs				
Not working	37168	0.420	0.416	0.425
0.0-9.9 hours	7236	0.090	0.087	0.093
10.0-19.9 hours	2731	0.033	0.031	0.035
20.0-29.9 hours	4125	0.048	0.047	0.050
30.0-39.9 hours	12328	0.149	0.146	0.152
40.0-49.9 hours	16550	0.203	0.199	0.207
50.0-59.9 hours	2791	0.032	0.030	0.033
60+ hours	2541	0.025	0.024	0.026

SOURCE: June 2020 LFS, N = 85,470 adults age 17 and older

NOTES: Estimates include survey-provided sample weights. Estimates provided as proportions unless otherwise specified.

Table D2 Descriptives Statistics for Employment Variables, May CPSS 2

	Sample Frequency	Proportion or Mean		
		Estimate	95% Confidence Interval	
			Lower	Upper
Labour force status				
Employed, at work	2227	0.463	0.437	0.488
Employed, absent from work (not due to COVID-19)	122	0.026	0.019	0.033
Employed, absent from work (due to COVID-19)	323	0.068	0.056	0.081
Not employed	1879	0.443	0.416	0.469
Might lose job in next 4 weeks (Employed respondents, N = 3455)				
Strongly agree	110	0.079	0.061	0.097
Agree	111	0.081	0.065	0.097
Neither agree nor disagree	261	0.204	0.175	0.234
Disagree	439	0.272	0.245	0.300
Strongly disagree	655	0.363	0.334	0.392
COVID-19 impact on ability to meet financial needs				
Major impact	344	0.090	0.074	0.106
Moderate impact	599	0.161	0.141	0.181
Minor impact	863	0.211	0.190	0.232
No impact	2235	0.429	0.403	0.454
Too soon to tell	510	0.109	0.093	0.125

SOURCE: May 2020 CPSS 2, N = 4,551 respondents age 15 and older

NOTES: Estimates include survey-provided sample weights Estimates provided as proportions unless otherwise specified.

Appendix References

Fredriksen-Goldsen, K. I., Kim, H. J., & Barkan, S. E. (2012). Disability among lesbian, gay, and bisexual adults: Disparities in prevalence and risk. *American Journal of Public Health,* 102(1), e16-e21.

Black, N., Johnston, D. W., & Suziedelyte, A. (2017). Justification bias in self-reported disability: New evidence from panel data. *Journal of Health Economics,* 54, 124-134.

Morris, S., Fawcett, G., Brisebois, L., & Hughes, J. (2018). A demographic, employment and income profile of Canadians with disabilities aged 15 years and over, 2017. Statistics Canada no. 89-654-X201800.

Statistics Canada. (2020a). *Census profile, 2016 Census.* Statistics Canada no. 98-316-X2016001. https://www12.statcan.gc.ca/census-recensement/2016/dp-pd/prof/index.cfm?Lang=E

Statistics Canada. (2020b.) Education Highlight Tables, 2016 Census. Statistics Canada Catalogue no. 98-402-X2016010. http://www12.statcan.gc.ca/census-recensement/2016/dp-pd/hlt-fst/edu-sco/index-eng.cfm

Waite, S., & Denier, N. (2019). A Research note on Canada's LGBT data landscape: Where we are and what the future holds. *Canadian Review of Sociology/Revue canadienne de sociologie,* 56(1), 93-117.
